# Feedback coordination of FoxO-mediated antibacterial immunity by PDGF/VEGF signaling establishes hemolymph microbiota homeostasis in shrimp

**DOI:** 10.1371/journal.ppat.1014307

**Published:** 2026-06-04

**Authors:** Ping-Ping Liu, Meng Zhang, Zhe Wei, Wei-Guang Wang, Jin-Xing Wang, Yuanning Li, Xian-Wei Wang

**Affiliations:** 1 School of Life Sciences, Shandong University, Qingdao, China; 2 State Key Laboratory of Microbial Technology, Shandong University, Qingdao, China; 3 Institute of Marine Science and Technology, Shandong University, Qingdao, China; National Institutes of Health, UNITED STATES OF AMERICA

## Abstract

A balanced immune response is required to limit the microbes without causing damage to the host. The forkhead box O (FoxO)-mediated immunity plays a pivotal role in maintaining microbiota homeostasis by regulating the expression of antimicrobial effectors in non-infected arthropods. However, the mechanism by which FoxO activity is appropriately coordinated remains unclear. In this study, we elucidated a feedback loop that coordinates FoxO-mediated antibacterial response using shrimp as a model. In this feedback loop, the commensal hemolymph microbiota maintains basal activation of FoxO, which determines the expression of antimicrobial effectors, platelet-derived growth factor/vascular endothelial growth factor (PDGF/VEGF)-related factor 1 (Pvf1) and PDGF/VEGF-related receptor 4 (Pvr4). This ligand-receptor system enhances phosphatidylinositol 3-kinase (PI3K)-protein kinase B (PKB/Akt) activity, limiting the excessive activation of FoxO and expression of antimicrobial effectors. This feedback loop is essential for maintaining the equilibrium of the microbiota, and its strength increases following a pathogenic infection, reducing the incidence of infection-induced mortality and tissue damage. This study revealed a microbiota-initiated feedback loop that balances FoxO-mediated antibacterial immunity for the establishment of microbiota homeostasis, and provides new insights into the functional diversification of PDGF/VEGF signaling.

## Author summary

FoxO-mediated transcription of antimicrobial effectors plays an important role in arthropod-bacteria interactions. This study reveals a feedback regulatory mechanism by which commensal microbiota initiates the basal activation of FoxO and expression of Pvf1/Pvr4, and Pvf1/Pvr4 system coordinates the strength of FoxO-mediated antibacterial immunity. A balanced immunity allows the successful colonization and persistence of commensal microbiota; therefore, this mechanism may contribute significantly to host health by facilitating the establishment of commensal microbiota homeostasis. Moreover, the findings that Pvf1 is an essential factor to maintain host health and to enhance host resistance against pathogenic infections may shed lights into the development of new approaches for disease prevention and control in aquaculture.

## Introduction

Many multicellular organisms host diverse microbial communities which primarily reside on their epithelial surfaces. Some microbiota even resides stably or temporarily in the hemolymph of arthropods, including insect and crustaceans [1–5]. In crustaceans, the open circulatory system may permit the establishment and maintenance of a low-abundance microbiota in the hemolymph under healthy conditions. The commensal microbiota plays pivotal roles in resisting infections, providing nutrients, promoting development, and influencing cognitive processes [6]. Disruption of the microbiota may cause the onset of chronic inflammatory diseases, such as autoimmunity, allergies, and metabolic syndrome. These conditions can eventually lead to abnormal development of organs or tissues or a shortened lifespan of the host [7,8]. Therefore, it is essential to maintain tight control over the microbiota to ensure homeostasis. This needs a fine-regulated host immunity which can limit the microbiota and simultaneously does not damage host.

The interaction between microbiota components and host immune system is of great consequence in determining the strength of the host immunity [9]. Two key signaling pathways, NF-κB and FoxO signaling, have been shown to be deeply involved in arthropod-bacterial interactions [10,11]. In *Drosophila*, peptidoglycan recognition proteins (PGRPs), particularly PGRP-LC and PGRP-LE, initiate the IMD signaling pathway upon detection of diaminopimelic (DAP)-type peptidoglycan (PGN) released by most gram-negative bacteria and some *Bacillus* species. This leads to nuclear translocation of the NF-κB family transcription factor Relish. Subsequently, the activated Relish protein induces the expression of antimicrobial peptides (AMPs), which effectively limit the commensal microbiota and neutralize infectious pathogens [12–14]. To prevent excessive immune activation, several negative regulators, including certain amidase PGRPs and poor IMD response upon knock-in (pirk) that are both target genes and inhibitors of the IMD/Relish pathway, act on its different nodes to achieve suppression [15–17].

Besides of IMD/Relish signaling, forkhead box O (FoxO) signaling, which primarily integrates signals to regulate metabolism and stress resistance [18,19], has also been demonstrated to play a crucial role in arthropod-bacterial interactions. In non-infected *Drosophila*, the generation of AMPs depends on FoxO but not on pathogen-responsive immunoregulatory pathways. FoxO binds directly to the regulatory region of the *Drosomycin* promoter, regulating its transcription. This regulatory process is further enhanced when non-infected animals are subjected to energy deficiencies or stress, enabling the host to modulate its defense responses against the microbiota to adapt to environmental changes [20]. In the context of pathogenic bacterial infections, particularly those affecting the oral cavity, FoxO signaling is markedly activated, directing the induction of AMPs, including *diptericin*, *attacinB*, and *attacinA*, and enhancing host resistance to infection, thus promoting survival [11]. In crustaceans, FoxO is also involved in commensal microbiota homeostasis and defense against pathogenic bacterial infection [21,22]. Given these findings, it can be concluded that FoxO plays a pivotal role in arthropod-bacterial interactions. However, the understanding of the coordination of FoxO-mediated antibacterial immunity remains largely limited.

In an attempt to identify the immune factors regulated by the commensal hemolymph microbiota in kuruma shrimp (*Marsupenaeus japonicus*), a platelet-derived growth factor/vascular endothelial growth factor (PDGF/VEGF)-related factor (Pvf1) and its receptor Pvr4 were both found down-regulated following microbiota elimination. The hemolymph microbiota is responsible for the basal expression of FoxO-mediated Pvf1 and Pvr4. Consequently, the Pvf1/Pvr4 system amplifies the PI3K-Akt pathway, inhibiting excessive FoxO activation. This feedback regulatory mechanism balances FoxO-mediated antibacterial immunity and ultimately results in the establishment of hemolymph microbiota homeostasis and resistance to exogenous pathogenic infection. Therefore, by revealing the Pvf1/Pvr4/FoxO feedback loop, this study provides insights into the coordinating regulation of FoxO-mediated antibacterial immunity and hemolymph microbiota homeostasis in arthropods.

## Results

### Hemolymph microbiota regulates the basal expression of Pvf1 and Pvr4

**Fig 1:**
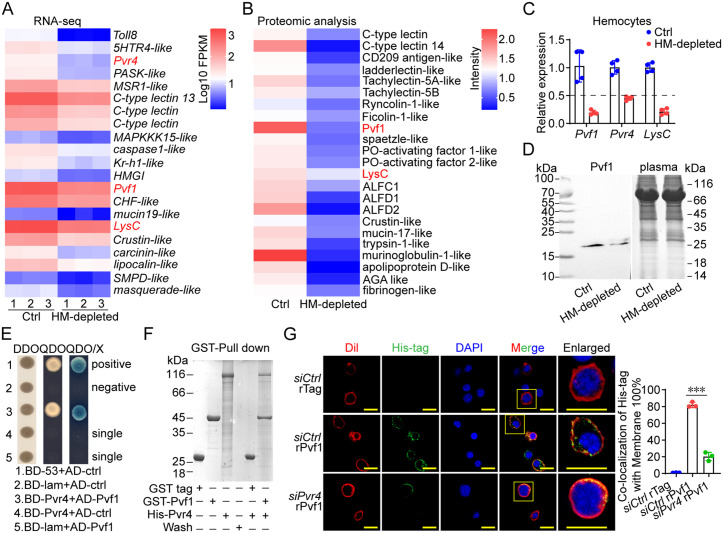
Identification of key molecules regulated by hemolymph microbiota. **(A-B)** Heat map showing genes and plasma protein whose expression levels were reduced after depletion of hemolymph microbiota. Shrimp were treated with antibiotics to generate hemolymph microbiota-depleted (HM-depleted) animals, while the Ctrl group shrimp received an equal volume of PBS injection. For hemocytes, gene expression levels are presented as Log10-transformed FPKM values from three independent biological replicates collected 3 d after antibiotics or PBS treatment **(A)**. For plasma proteins, the average signal intensities from three independent replicates collected at the same time point are shown **(B)**. **(C-D)** Hemolymph microbiota depletion reduces the mRNA levels of *Pvf1*, *Pvr4*, and *LysC* and the plasma protein level of Pvf1. The mRNA expression of *Pvf1*, *Pvr4*, and *LysC* in hemocytes were quantified by qRT-PCR **(C)**. The plasma protein level of Pvf1 were analyzed by western blotting, with equal protein loading confirmed by Coomassie Brilliant Blue staining **(D)**. **(E)** Interaction between Pvf1 and Pvr4 analyzed by yeast two-hybrid (Y2H) assay. Transformants were grown on DDO (Leu-, Trp-), QDO (Ade-, Leu-, Trp-, His-), and QDO/X (Ade-, Leu-, Trp-, His-, X-gal+) selective media. AD, activating domain; BD, binding domain. **(F)** Direct interaction between Pvf1 and Pvr4 verified by GST pull-down assay. GST-Pvf1 or GST-tagged control (GST-tag) was incubated with His-Pvr4, followed by precipitation using GST resin. After washing the resin with PBS, the bound proteins were eluted using elution buffer. Input recombinant proteins (GST-tag, GST-Pvf1, and His-Pvr4) are shown in lanes 1-3, and the final wash and elution solution are shown in lanes 4-6 after SDS-PAGE and Coomassie Brilliant Blue staining. **(G)** Pvf1 association with the hemocyte surface is significantly reduced in Pvr4-silenced hemocytes. Hemocytes isolated from Pvr4-silenced shrimp were incubated with His-tagged recombinant Pvf1 (rPvf1) or control protein (rTag). Surface-associated rPvf1 was detected by a primary antibody targeting the His-tag followed by a DyLight 488-conjugated secondary antibody. Nuclei (DAPI, blue), rPvf1 (DyLight 488, green), and cell membranes (DiI, red) are shown. Scale bar = 5 μm. Quantification was performed from three randomly selected fields. rPvf1 binding was normalized to the rTag control, and membrane colocalization was calculated as the percentage of hemocytes showing rPvf1-membrane overlap. Statistical analysis was performed using the Student’s *t-test*. ***p < 0.001. Images represent three replicates.

Commensal microbiota regulate host immune responses systemically and locally [23]. To identify immune-related molecules controlled by the hemolymph microbiota, we depleted hemolymph-associated bacteria by injecting antibiotics into the shrimp hemocoel. Plate counting and quantitative PCR (qPCR) analyses revealed that hemolymph culturable and total bacterial loads significantly reduced following antibiotics treatment, collectively demonstrating the successful establishment of the hemolymph microbiota-depleted (HM-depleted) shrimp model (S1 Fig). To screen for host factors regulated by the hemolymph microbiota, total RNA from hemocytes and plasma proteins were collected for transcriptomic (RNA-seq) and proteomic analyses, respectively. The presence of lysozyme C (LysC), which had been proven to function as a core antimicrobial factor involved in shrimp microbiota homeostasis through both direct antibacterial activity and coordination with other immune effectors acting on bacteria [21,24], in both datasets confirmed screening validity (Fig 1A and 1B).

Notably, a PDGF/VEGF family member, Pvf1, was also consistently down-regulated at both the transcript and protein levels following microbiota depletion. Members of the Pvf family are synthesized and secreted to perform extensive biological functions by interacting with their corresponding receptor [25]. Concurrently, the transcription of *Pvr4*, which encodes a potential receptor for the Pvf family, was significantly reduced in hemolymph microbiota-depleted shrimp (Fig 1A). To validate the screening results, we quantified the expression of *Pvf1*, *Pvr4*, and *LysC* by qRT-PCR and confirmed that their mRNA levels were markedly decreased after microbiota depletion (Fig 1C). Moreover, the blotting results also revealed a corresponding reduction in Pvf1 protein level in the plasma of microbiota-depleted shrimp (Fig 1D).

To assess the functional relevance of the co-occurrence of Pvf1 and Pvr4, we performed yeast two-hybridization and pull-down analyses to detect whether Pvf1 and Pvr4 would form a signaling complex. The results demonstrated a direct interaction between Pvf1 and the extracellular ligand-binding domain of Pvr4 (Fig 1E and 1F). Furthermore, immunofluorescence assays showed that recombinant Pvf1 (rPvf1) could attach to the hemocyte cytomembrane; however, this attachment was substantially impaired upon *Pvr4* knockdown (Fig 1G). Collectively, these results indicate that the hemolymph microbiota sustains the baseline expression of a functional Pvf1/Pvr4 system.

### Hemolymph microbiota regulates Pvf1/Pvr4 system through FoxO

**Fig 2:**
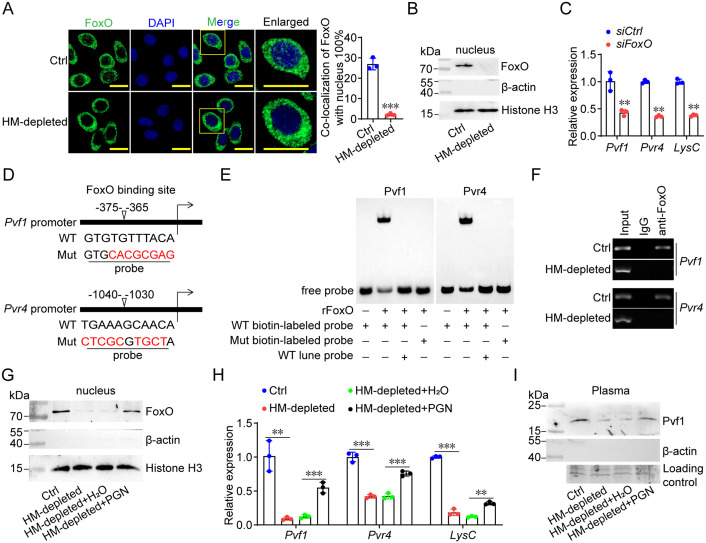
Hemolymph microbiota regulates the expression of Pvf1 and Pvr4 through FoxO. (A-B) Induction of FoxO nuclear localization by the hemolymph microbiota. Hemocytes were collected from HM-depleted and control shrimp. Immunocytochemical analysis was performed to examine the subcellular localization of FoxO. Scale bar = 10 μm. FoxO nuclear localization was quantified from three randomly selected fields using ImageJ software (A). FoxO nuclear levels were further analyzed by blotting assay. Histone H3 and β-actin were used as internal references for nuclear and cytoplasmic proteins, respectively (B). (C) Identification of FoxO-dependent expression of *Pvf1*, *Pvr4*, and *LysC*. The mRNA levels of *Pvf1*, *Pvr4*, and *LysC* in hemocytes were quantified by qRT–PCR after knockdown of *FoxO*. (D) The Presence of putative FoxO-binding sites in the promoters of *Pvf1* and *Pvr4*. Promoter sequences were obtained from the *M. japonicus* genome (GenBank accession numbers GCA_017312705.1, GCA_002291165.1). Potential elements were analyzed using the online PROMO 3.0 and JASPAR tools. WT: wild-type probes; Mut: mutated probes. (E) Binding of rFoxO to oligonucleotides derived from the *Pvf1* or *Pvr4* promoters revealed by electrophoretic mobility shift assay (EMSA). Biotin-labeled wild-type or mutated oligonucleotide probes (5 ng) were incubated with recombinant FoxO protein (rFoxO, 2 μg; lanes 2 and 4). Control reactions contained rTag instead of rFoxO (lane1). Competition assays were performed in the presence of excess unlabeled wild-type probes (500 ng) (lane3). DNA-protein complexes were resolved on 6% native polyacrylamide gels and detected using a chemiluminescent biotin-labeled nucleic acid detection system. (F) Binding of FoxO to the *Pvf1* and *Pvr4* promoters is enhanced by hemolymph microbiota. Hemocytes were collected from HM-depleted and control shrimp 3 d after antibiotics or PBS treatment and subjected to chromatin immunoprecipitation (ChIP) assays. Immunoprecipitates containing FoxO-binding sites were detected by RT-PCR using specific primers. (G-I) Rescue of FoxO nuclear localization and Pvf1, Pvr4, and LysC expression in HM-depleted shrimp by PGN. HM-depleted shrimp were injected with PGN, while control shrimp received an equal volume of H_2_O. FoxO nuclear levels were analyzed at 6 h (G); mRNA levels of *Pvf1*, *Pvr4* and *LysC* were quantified at 12 h (H); and plasma Pvf1 protein levels were analyzed at 12 h (I). Statistical analysis was performed using the Student’s *t-test*. ** *p* < 0.01, ***p < 0.001 and ns, not significant. Images represent three replicates.

Previous studies have shown that a key transcription factor, FoxO, is activated by commensal microbiota and controls the basal expression of multiple immune factors, including *LysC*, which are critical for microbiota homeostasis in shrimp [21,22]. Based on these findings, we investigated whether FoxO is involved in the hemolymph microbiota-mediated regulation of the Pvf1/Pvr4 system. Immunocytochemical and immunoblotting analyses revealed that FoxO nuclear levels were markedly reduced in hemolymph microbiota-depleted shrimp (Fig 2A-2B). Consistently, knockdown of *FoxO* resulted in a significant decrease in the expression of *Pvf1* and *Pvr4* (Fig 2C). These data indicate the possible involvement of FoxO in the regulation of the Pvf1/Pvr4 system.

To determine whether FoxO directly regulates *Pvf1* and *Pvr4* transcription, we analyzed their promoter regions and identified putative FoxO-responsive elements in both genes (Fig 2D). An electrophoretic mobility shift assay (EMSA) was therefore performed to determine whether FoxO interacts with these elements. The results demonstrated direct binding of recombinant FoxO (rFoxO) to wild-type, but not mutated oligonucleotide probes (Fig 2E). In parallel, chromatin immunoprecipitation (ChIP) assays showed the presence of *Pvf1* and *Pvr4* promoter fragments containing FoxO-responsive elements in FoxO immunoprecipitates. Notably, depletion of the hemolymph microbiota led to the disappearance of promoter fragments in ChIP assay, suggesting the necessity of hemolymph microbiota for FoxO-mediated *Pvf1* and *Pvr4* transcription (Fig 2F). Collectively, these data supported that microbiota-maintained expression of the Pvf1/Pvr4 system is mediated by FoxO.

We next examined whether microbiota-derived signals contribute to FoxO-mediated regulation of the Pvf1/Pvr4 system. As we had previously revealed that microbiota-derived PGN fragments (muropeptides) activates FoxO-mediated antibacterial immunity in shrimp, we next verified the role of PGN [21]. We supplemented microbiota-depleted shrimp with PGN to assess its effect on FoxO activity. As shown in Fig 2G, while microbiota elimination impaired FoxO nuclear localization, PGN replenishment restored FoxO nuclear accumulation. Consistent with this restoration, PGN replenishment also restored the expression of the *Pvf1*, *Pvr4*, and *LysC*, as well as the plasma level of Pvf1 (Fig 2H and 2I). Together, these results further proved that hemolymph microbiota is responsible for the FoxO-mediated regulation of Pvf1/Pvr4 system.

### Pvf1/Pvr4 system facilitates the persistence and homeostasis of hemolymph microbiota

**Fig 3:**
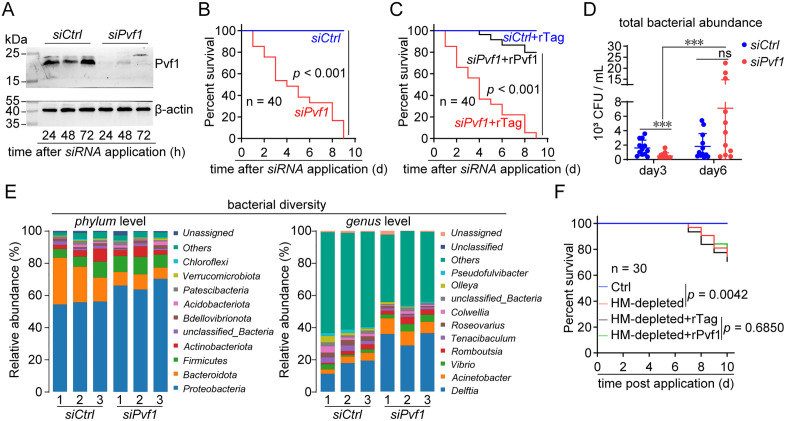
Function of Pvf1 in maintaining hemolymph microbiota persistence and homeostasis. **(A)** RNAi efficiency of Pvf1. Pvf1 protein levels in hemocytes were detected by western blotting at the indicated times points after siRNA (5 μg/g) treatment. siCtrl served as the control. **(B)** Survival of shrimp following *Pvf1* knockdown. Shrimp mortality was recorded daily after siRNA application. **(C)** Survival of shrimp after *Pvf1* knockdown followed by rPvf1 supplementation. Shrimp were injected with rPvf1 (2 μg) after *Pvf1* silencing, while control shrimp received an equal amount of rTag. Mortality was recorded daily. **(D)** Total bacterial abundance in the hemolymph after *Pvf1* knockdown. Hemolymph was collected 3 and 6 d after siRNA treatment. Total DNA was extracted, and bacterial load was quantified by qPCR targeting 16S rDNA. Bacterial abundance was calculated as colony-forming units per milliliter hemolymph (CFU/mL) based on a standard curve. In scatter plots, each dot represents one shrimp. **(E)** Variation in hemolymph microbiota composition after *Pvf1* knockdown. Shrimp hemolymph samples were collected 6 d after siRNA treatment and analyzed by 16S rRNA gene high-throughput sequencing at the phylum and genus levels. Each sample originated from at least 10 animals. Three biological replicates were performed. **(F)** Effects of rPvf1 on shrimp survival under hemolymph microbiota-depleted conditions. HM-depleted shrimp were generated by antibiotic treatment, followed by administration of rPvf1 (2 μg) or rTag. Shrimp mortality was recorded daily. Survival rate analysis was performed using the log-rank (Mantel-Cox) test, and other statistical analyses were performed using Mann-Whitney U test. *p < 0.05 and ***p < 0.001.

We have shown that hemolymph microbiota sustains the basal expression of Pvf1 and Pvr4. However, the functional role of Pvf1/Pvr4 system in host-microbiota interactions remained unclear. To clarify the biological significance of this ligand-receptor system, *Pvf1* expression was silenced by RNAi (Fig 3A) to determine whether there would be any consequence. Strikingly, Knockdown of *Pvf1* alone was sufficient to induce significant shrimp mortality in the absence of exogenous infection (Fig 3B). As Pvf1 is soluble in plasma, we used rPvf1 for exogenous supplementation to further clarify the function of Pvf1 in *vivo* (S2 Fig). We next assessed whether exogenous supplementation could rescue this phenotype. rPvf1 was injected into the shrimp hemocoel following *Pvf1* knockdown. As shown in Fig 3C, administration of rPvf1 markedly rescued shrimp mortality caused by *Pvf1* knockdown, indicating that Pvf1 is essential for shrimp survival under basal conditions. We next checked whether the increased mortality observed after *Pvf1* knockdown was associated with alterations in hemolymph microbiota homeostasis. The data showed that the total bacterial abundance in the hemolymph changed dramatically. Specifically, the bacterial abundance decreased sharply at 3 days after *Pvf1* knockdown, followed by an uncontrolled surge by day 6 (Fig 3D). We also found that rPvf1 administration restored the hemolymph microbiota homeostasis in *Pvf1* knockdown shrimp (S2 Fig). A microbial diversity analysis was next performed to reveal the change of microbiota composition and features. As shown in Fig 3E, *Proteobacteria*, *Bacteroidota* and *Firmicutes* were the dominant phyla in the shrimp hemolymph microbiota. Following *Pvf1* knockdown, the relative abundances of these phyla were markedly altered: *Bacteroidota* was substantially reduced, whereas *Proteobacteria* and *Firmicutes* were significantly increased. At the genus level, the abundances of opportunistic taxa such as *Delftia*, *Acinetobacter*, and *Vibrio* were significantly elevated. In contrast, taxa previously associated with beneficial host-microbe interactions, including *Endozoicomonas*, *OM43_clade* and *Phaeobacter*, became completely undetectable in the *Pvf1* knockdown group [26,27]. Together, these results suggest that Pvf1 is essential for the persistence and homeostasis of the hemolymph microbiota.

To distinguish whether the effect of Pvf1 on shrimp survival was direct or mediated through the hemolymph microbiota, we next assessed shrimp survival under hemolymph microbiota-depleted conditions. Removal of the hemolymph microbiota significantly, but incompletely, reduced shrimp survival, demonstrating that microbial integrity contributes to host viability. Importantly, supplementation with exogenous rPvf1 failed to rescue survival in hemolymph microbiota-depleted shrimp, in which endogenous Pvf1 expression was already suppressed (Fig 3F). Collectively, these findings support a model in which Pvf1 promotes shrimp survival precisely by maintaining a functional and balanced hemolymph microbiota, rather than acting as a direct survival factor.

### Pvf1/Pvr4 system limits FoxO-mediated antibacterial immunity

**Fig 4:**
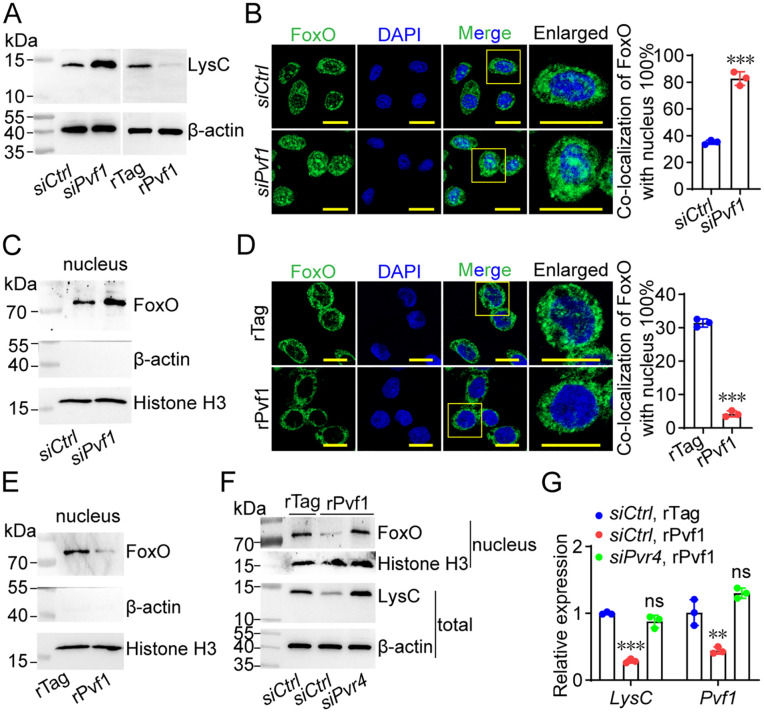
The regulatory effect of Pvf1/Pvr4 system on FoxO transcriptional activity. **(A)** Effects of Pvf1 on LysC protein expression. LysC protein levels in hemocytes were analyzed 24 h after *Pvf1* knockdown via siRNA treatment or 24 h after rPvf1 (2 μg) treatment. **(B)** Induction of FoxO nuclear localization following *Pvf1* knockdown. Hemocytes were collected 24 h after siRNA treatment and subjected to immunocytochemical analysis. Scale bar = 10 μm. FoxO nuclear localization was quantified from three randomly selected fields using ImageJ software. **(C)** FoxO nuclear levels after *Pvf1* knockdown. Nuclear proteins were isolated from hemocytes 24 h after siRNA treatment and analyzed by blotting assay. **(D-E)** Inhibition of FoxO nuclear localization by rPvf1 treatment. Hemocytes were sampled 12 h after rPvf1 treatment for immunocytochemical analysis and quantification of FoxO nuclear colocalization **(D)**, as well as blotting assay of FoxO nuclear levels **(E)**. Scale bar = 10 μm. **(F)** Loss of rPvf1-mediated inhibition of FoxO nuclear levels and LysC protein expression following *Pvr4* knockdown. After *Pvr4* silencing, shrimp were injected with rPvf1 or rTag. Nuclear proteins (12 h) and total proteins (24 h) were isolated from hemocytes, and FoxO nuclear levels and LysC protein levels were analyzed by blotting assay. **(G)** Loss of rPvf1-mediated inhibition of *LysC* and *Pvf1* transcription following *Pvr4* knockdown. After Pvr4 silencing, shrimp were injected with rPvf1 or rTag. Hemocytes were collected 24 h after rPvf1 treatment, and mRNA levels of *LysC* and *Pvf1* were quantified by qRT-PCR. Statistical analysis was performed using the Student’s *t-test*. ** *p* < 0.01, ***p < 0.001 and ns, not significant. Images represent three replicates.

Next, we investigated the mechanism by which Pvf1 contributes to hemolymph microbiota homeostasis. Effective control of microbial populations requires a balanced immune response that restricts bacterial expansion without causing excessive host damage. Given that the key antimicrobial factor LysC—whose expression is primarily mediated by the transcription factor FoxO (Fig 2C) [21]—is also maintained by the hemolymph microbiota, we hypothesized that Pvf1 modulates FoxO-mediated antibacterial immunity. To test this hypothesis, we then suppressed or enhanced Pvf1 activity by RNAi-mediated knockdown or rPvf1 injection, respectively, to assess its role in FoxO-mediated antibacterial immunity. Indeed, *Pvf1* knockdown significantly increased, whereas rPvf1 administration decreased, LysC protein levels (Fig 4A). Consistent with this, immunocytochemical and immunoblotting analyses revealed enhanced FoxO nuclear accumulation following *Pvf1* knockdown, while rPvf1 administration suppressed FoxO nuclear localization (Fig 4B-4E). As shown in Fig 2G, PGN replenishment not only induced FoxO activation, but also restored Pvf1 expression, suggesting that PGN-induced FoxO nuclear translocation may in turn be restrained by the induced Pvf1 expression. To test this possibility, we further stimulated shrimp with PGN after *Pvf1* knockdown. The results showed that knockdown of *Pvf1* further enhanced PGN-induced FoxO nuclear localization (S3 Fig). Similar results were also observed when the native Pvf1 protein was neutralized by a specific Pvf1 antibody (S4 Fig). These results indicate that Pvf1 negatively regulates FoxO-mediated antibacterial immunity by restricting FoxO nuclear localization.

To determine whether the effect of Pvf1 FoxO activity depended on its receptor, we administered rPvf1 to shrimp in which *Pvr4* had been pre-silenced. The blotting results showed that the ability of rPvf1 to reduce FoxO nuclear levels and suppress LysC protein level was abolished upon *Pvr4* knockdown (Fig 4F), confirming the dependence of Pvf1 function on Pvr4. Intriguingly, we found that the expression of both *Pvf1* and *Pvr4* is itself regulated by microbiota-activated FoxO signaling (Fig 2C-2F), yet is subsequently suppressed following activation of Pvf1/Pvr4 system by rPvf1 administration (Fig 4G). Taken together, these results reveal a coherent feedback loop: the hemolymph microbiota activates FoxO, which upregulates both antimicrobial effectors (e.g., LysC) and the Pvf1/Pvr4 system; in turn, Pvf1/Pvr4 signaling dampens FoxO activity, thereby tempering antimicrobial immunity to permit microbiota persistence and homeostasis.

### Pvf1/Pvr4 system limits FoxO-mediated antibacterial immunity through enhancing PI3K/Akt signaling

**Fig 5:**
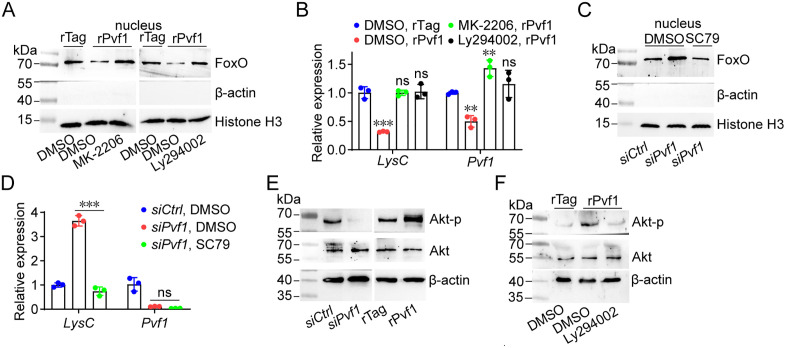
Pvf1/Pvr4 system inhibits FoxO transcriptional activity through activation of the PI3K-Akt pathway. **(A)** Loss of rPvf1-mediated inhibition of FoxO nuclear levels upon blockade of PI3K-Akt pathway. Shrimp were pretreated with the Akt inhibitor MK-2206, the PI3K inhibitor Ly294002 or DMSO for 2 h, followed by rPvf1 injection. FoxO nuclear levels in hemocytes were analyzed by blotting assay 12 h after rPvf1 administration. **(B)** Loss of rPvf1-mediated inhibition of *LysC* and *Pvf1* transcription upon blockade of PI3K-Akt pathway. Shrimp were pretreated with MK-2206, Ly294002 or DMSO for 2 h, followed by rPvf1 injection. Hemocytes were collected 24 h after rPvf1 injection, and mRNA levels of *LysC* and *Pvf1* were quantified by qRT-PCR. **(C)** Reversal of the effects of *Pvf1* knockdown on FoxO nuclear levels by Akt activation. After *Pvf1* silencing, shrimp were injected with the Akt activator SC79 or DMSO. FoxO nuclear levels in hemocytes were analyzed by blotting assay 2 h after injection. **(D)** Reversal of *Pvf1* knockdown-induced *LysC* and *Pvf1* transcription by Akt activation. After *Pvf1* silencing, shrimp were injected with SC79 or DMSO. Hemocytes were collected 12 h after injection, and mRNA levels of *LysC* and *Pvf1* were quantified by qRT-PCR. **(E)** Regulation of Akt phosphorylation by Pvf1. Phosphorylated Akt (Akt-p) and total Akt levels in hemocytes were analyzed by western blotting 24 h after *Pvf1* knockdown or 12 h after rPvf1 (2 μg) treatment. **(F)** Loss of rPvf1-mediated Akt phosphorylation following PI3K inhibition. Shrimp were pretreated with Ly294002 or DMSO for 2 h, followed by rPvf1 injection. Levels of phosphorylated Akt and total Akt protein levels were analyzed by western blotting 12 h after rPvf1 administration. Statistical analysis was performed using the Student’s *t-test*. ** *p* < 0.01, ***p < 0.001 and ns, not significant. Images represent three replicates.

Next, we investigated the signaling mechanism by which the Pvf1/Pvr4 system limits FoxO-mediated antibacterial immunity. Pvr4 is a type I single-transmembrane receptor tyrosine kinase containing an intracellular domain for tyrosine protein kinase activity, and that PI3K/Akt signaling—a canonical downstream pathway of receptor tyrosine kinase activation—is a well-established negative regulator of FoxO activity [28,29]. Based on this, we examined whether PI3K/Akt signaling mediates Pvf1/Pvr4-dependent regulation of FoxO. Indeed, pharmacological inhibition of either Akt or PI3K abolished the ability of rPvf1 to suppress FoxO transcriptional activity. Pretreatment with the Akt inhibitor MK-2206 or the PI3K inhibitor LY294002 abolished rPvf1-induced suppression in FoxO nuclear leevels (Fig 5A) and *LysC* expression (Fig 5B). Conversely, the Akt activator SC79 suppressed the increase in FoxO nuclear accumulation (Fig 5C) and *LysC* expression (Fig 5D) caused by *Pvf1* knockdown. These pharmacological data position PI3K/Akt signaling downstream of the Pvf1/Pvr4 system and upstream of FoxO, supporting a sequential Pvf1/Pvr4/FoxO signaling axis.

We next further examined the role of Pvf1 in regulating Akt activity. As shown in Fig 5E, Pvf1 knockdown decreased, whereas rPvf1 administration increased, Akt phosphorylation, respectively, suggesting that Pvf1 indeed affects Akt kinase activity. Importantly, the rPvf1-induced Akt phosphorylation was blocked by pretreatment with the PI3K inhibitor Ly294002 (Fig 5F), indicating that Pvf1 activates Akt in a PI3K-dependent manner. Taken together, these results demonstrate that the Pvf1/Pvr4 system restrains FoxO-mediated antibacterial immunity by enhancing PI3K/Akt signaling, thereby suppressing FoxO transcriptional activity and downstream antimicrobial effector expression.

### Pvf1/Pvr4 system protects shrimp against V. parahaemolyticus infection

**Fig 6:**
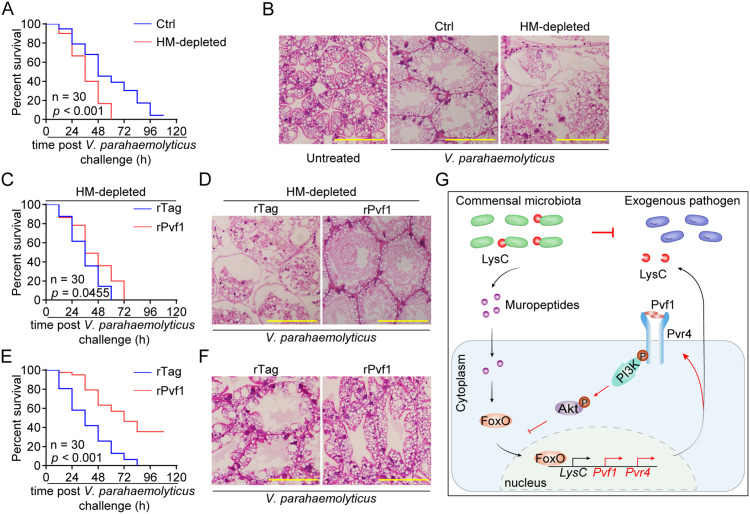
Pvf1 enhances shrimp resistance to *V. parahaemolyticus* infection. **(A)** Exacerbation of mortality in HM-depleted shrimp following *V. parahaemolyticus* infection. HM-depleted and control shrimp were generated as described above and subsequently immersed in seawater containing V. parahaemolyticus. Shrimp mortality was recorded every 12 **h. (B)** Enhanced hepatopancreatic damage in HM-depleted shrimp after *V. parahaemolyticus* infection. HM-depleted shrimp were soaked in seawater containing *V. parahaemolyticus*. Hepatopancreatic tissues were collected 2 d after infection, sliced, and stained with H&E. Scale bar = 200 μm. Untreated shrimp without infection were included as controls. (C) rPvf1 partially rescues mortality of HM-depleted shrimp after *V. parahaemolyticus* infection. HM-depleted shrimp supplemented with or without rPvf1 were soaked in seawater containing *V. parahaemolyticus*. Shrimp death was recorded every 12 **h.** (D) rPvf1 alleviates hepatopancreatic damage in HM-depleted shrimp after *V. parahaemolyticus* infection. HM-depleted shrimp supplemented with or without rPvf1 were soaked in seawater containing *V. parahaemolyticus*. Hepatopancreatic tissues were collected 2 d after infection, sliced, and stained with H&E. Scale bar = 200 μm. (E) rPvf1 reduces mortality of normal shrimp after *V. parahaemolyticus* infection. After 2 d of rPvf1 or rTag treatment, shrimp were soaked in seawater containing *V. parahaemolyticus*. Shrimp survival rate was recorded every 12 **h.** (F) rPvf1 mitigates hepatopancreatic damage in normal shrimp after *V. parahaemolyticus* infection. After 2 d of rPvf1 or rTag treatment, shrimp were soaked in seawater containing *V. parahaemolyticus*. Hepatopancreatic tissues were collected 2 d after infection, sliced, and stained with H&E. Scale bar = 200 μm. **(G)** Model of the Pvf1/Pvr4/FoxO negative feedback loop. Shrimp were challenged by immersion in seawater containing *V. parahaemolyticus* at a final concentration of 1 × 10^6^ CFU/mL. The survival data represent two independent replicates. Survival assay was analyzed by log-rank (Mantel-Cox) test. Images represent three replicates.

We have revealed that the Pvf1/Pvr4 system contributes to microbiota homeostasis by limiting the microbiota-activated and FoxO-mediated antibacterial immunity. As both Pvf1 and Pvr4 expression were upregulated by *Vibrio parahaemolyticus* infection, and this induction disappeared after *FoxO* knockdown (S5 Fig), we next studied the significance of this feedback regulatory mechanism in host resistance to *V. parahaemolyticus*, a serious pathogen to shrimp aquaculture [30]. Consistent with a protective role of the hemolymph microbiota, depletion of hemolymph microbiota also led to an increased susceptibility to bacterial infection and higher mortality than in the control group (Fig 6A). The hepatopancreatic morphology was also monitored. In healthy shrimp, hepatopancreatic glandular tubules were regularly arranged and well-defined. In contrast, *V. parahaemolyticus* infection caused pronounced swelling of the tubular lumen and detachment of epithelial cells. These pathological alterations were markedly exacerbated in hemolymph microbiota-depleted shrimp, which exhibited severe erosion of glandular tubule (Fig 6B). Additional application of rPvf1, which is able to promote the microbiota establishment, to microbiota-depleted shrimp relieved shrimp death (Fig 6C) and mitigated the hepatopancreatic damage (Fig 6D).

Thereafter, we investigated whether overexpression of Pvf1 in healthy shrimp could further enhance host disease resistance. As shown in Fig 6E and F, rPvf1 application rescued shrimp death and weakened hepatopancreatic damage caused by *V. parahaemolyticus* infection. The effect of rPvf1 applied in the presence of microbiota (Fig 6E) was stronger than that in the absence of microbiota (Fig 6C), indicating that the Pvf1-microbiota interaction is needed to enhance host disease resistance. Collectively, these data suggest that the hemolymph microbiota confers host resistance to external pathogenic infections, and that Pvf1 makes a considerable contribution to this resistance by regulating the persistence and homeostasis of the microbiota.

## Discussion

The Pvf1/Pvr4/FoxO regulatory loop represents a novel mechanism for balancing host antibacterial immunity. Feedback regulation is essential to generate an appropriate immunity. In the *D. melanogaster* model, feedback coordination of IMD/Relish-mediated antibacterial immunity was achieved through the involvement of two classes of molecules. One is the amidase PGRP family, which is synthesized under the regulation of the IMD pathway, and can cleave peptidoglycans, thereby reducing the levels of immunostimulatory components in the gut lumen [17,31–33]. Another is the negative regulatory factor Pirk, which disrupts and limits IMD signal transduction by targeting the intracellular components of the IMD pathway [16]. In shrimp, we have previously identified a regulatory mode which ensures that the basal antibacterial immunity is not overactivated. Lysozyme C (LysC) cleaves the microbiota peptidoglycan into muropeptides which effectively activate the FoxO-mediated antibacterial immunity. FoxO regulates the synthesis of LysC and a C-type lectin (Ctl24). By antagonizing the processing of peptidoglycan by LysC and preventing excessive amplification of the muropeptide-stimulated immune response, Ctl24 functions as a feedback factor to maintain the FoxO-mediated antibacterial immunity at an appropriate level [21]. This regulatory mechanism is analogous to the PGRP-mediated feedback mechanism in *D. melanogaster*. In this study, we discovered that Pvf1/Pvr4 system represents another feedback regulatory mode distinct from Ctl24, specifically in the regulation of FoxO activity. It is noteworthy that this mode resembles the *D. melanogaster* Pirk-related mode. Therefore, the current finding provides new insights into the mechanisms that balancing antibacterial immunity in arthropods.

The involvement of Pvf1/Pvr4 system in commensal microbiota homeostasis demonstrates the functional diversification of Pvf family. The typical role of Pvf family is to regulate development-related events. For example, vertebrate Pvf family members regulate angiogenesis, cell proliferation, migration, survival, and vascular permeability during angiogenesis [34]. In contrast, research on invertebrates has shown these family members induce blood cell proliferation in larvae and control blood cell migration [35–37]. Besides of the typical roles, this family also plays a pivotal role in immunity, particularly in regulating the intensity of the host immune response during infection, to prevent the detrimental effects of excessive immune activation. In murine macrophages, bacterial infection induces the expression of *VEGFR-3* and its ligand *VEGF-C* through activating TLR4-NF-κB signaling pathway. Conversely, VEGF-C/VEGFR-3 inhibits the overactivation of TLR4-NF-κB, preventing the risk of sepsis caused by endotoxic shock [38]. In *Drosophila*, Pvf2 plays a role in coordinating humoral and cellular immune responses, helping maintain equilibrium during infection [39]. In crustaceans, VEGF-family signaling has also been shown to respond to bacterial or viral challenge and to regulate the expression of immune effectors involved in pathogen defense [40–43]. In this study, we prove that the Pvf1 is able to suppress the excessive activation of shrimp antibacterial immunity, similar to those counterparts in mammals and *Drosophila*. However, this Pvf1/Pvr4-mediated mechanism has been elucidated in the context of microbiota homeostasis, but not in the context of pathogenic infection. Therefore, this study underscores a novel function for Pvf family and expands the knowledge about their significance. Given the conservation of the Pvf family, from invertebrates to vertebrates, it is plausible that its importance in regulating microbiota homeostasis may be conserved in other animals.

The finding that Pvf1/Pvr4 system contributes to microbiota homeostasis and resistance to pathogenic infections provided further evidence for the significance of hemolymph microbiota for shrimp health. Commensal microbiota contributes to host health by maintaining the expression of the Pvf family. For example, germ-free mice exhibit reduced neurogenesis compared with specific pathogen-free control mice. This is because germ-free mice have an impaired indole-AHR signaling pathway, resulting in a decreased expression of VEGF-α, which promotes different stages of adult neurogenesis [44,45]. In addition, the microbiota also enables host to resist pathogens by inducing the expression of Pvf family members. In *D. melanogaster*, intestinal commensal *Acetobacter pomorum* primes the NF-κB-dependent induction of a secreted factor, Pvf2, which combines with its receptor, PVR, and stimulates antiviral immunity. A deficiency in the intestinal microbiota renders the host more vulnerable to enteric viral infections [46]. In the present study, we show that the shrimp Pvf1/Pvr4 system maintains hemolymph microbiota homeostasis and thereby enhances host resistance to bacterial infection, further highlighting the mutually beneficial relationship between the host and its microbiota. Importantly, these findings argue that the hemolymph microbiota is not passively tolerated, but actively maintained in a controlled state that benefits host health. In this context, the Pvf1/Pvr4 system provides a mechanistic basis for such control by restraining excessive antibacterial immunity and thereby preventing over-elimination of the microbiota. This study revealed that shrimp Pvf1/Pvr4 system is able to maintain microbiota homeostasis and to enhance host resistance to infections, and again supported the deep involvement of Pvf family in the positive and mutually beneficial relationship between host and microbiota. As hemolymph microbiota is the key driving factor for the basal level of Pvf1/Pvr4 system, this study therefore clarified a new mechanism by which the microbiota confers benefits for host well-being.

The discovery that the Pvf1/Pvr4 system hinders FoxO nuclear level by increasing Akt phosphorylation expands the knowledge regarding FoxO regulation. In vertebrates, VEGF enhances tyrosine phosphorylation of VEGFR subsequently activating the PI3K/Akt axis [47], which is consistent with Pvf1/Pvr4-mediated PI3K/Akt activation in this study. However, the events downstream of Akt signaling vary from vertebrates to shrimp. In mammals, VEGFR3-activated Akt restrains NF-κB-driven immunity by upregulating the negative regulator SOCS1 [38]. In contrast, Pvf1/Pvr4-activated Akt in this study directly inhibits FoxO-mediated antibacterial immunity. As a pivotal transcription factor that responds to stress, FoxO undergoes a range of post-translational modifications, including phosphorylation and acetylation. Under typical circumstances, FoxO is phosphorylated by Akt, which makes it challenging to locate it in nucleus and interact with the promoter region of the target genes. In response to stress, inactivation of Akt results in the translocation of FoxO from the cytoplasm to the nucleus, where it binds to the corresponding promoter regions and initiates downstream gene expression [48]. A genome-wide RNAi screening of kinases and phosphatases that regulate FoxO activity in *Drosophila* had demonstrated that PVR inhibits FoxO entry into the nucleus. However, the mechanism by which PVR inhibits FoxO nuclear localization and the physiological significance of PVR in this process remains unclear [49]. In this study, we demonstrated that Pvf1, with Pvr4, activates the PI3K/Akt signaling pathway to inhibit FoxO nuclear localization and thereby FoxO-mediated transcription of antibacterial genes. This study revealed a sequential regulatory mechanism by which FoxO activity is regulated to prevent excessive FoxO activation. At the same time, these findings also raise a further mechanistic question as to how activated Pvr4 is coupled to PI3K/Akt at the receptor-proximal level. In RTK signaling, class IA PI3K is classically recruited through its p85 regulatory subunit, whose SH2 domains recognize phosphotyrosine-containing docking sites on activated receptors, as well established for PDGFR [50–52]. By contrast, VEGF-family receptors can couple to PI3K either through direct interaction or through adaptor-associated signaling modules. Direct association with PI3K has been reported for VEGFR-3 [38,53], whereas VEGFR-2-dependent PI3K/Akt activation more often involves intermediates such as Gab1 (Grb2-adapter binder 1), TSAd, and Src family kinases (SFKs) [54,55]. Notably, sequence analysis indicates that the shrimp PI3K p85 regulatory subunit contains conserved SH2 domains. Against this background, direct recruitment of a p85-containing PI3K complex by activated Pvr4 represents a plausible working model, although adaptor-assisted coupling cannot be excluded by our current data. Together, these observations further support the placement of Pvf1/Pvr4 upstream of PI3K/Akt, although the precise mechanism by which activated Pvr4 engages this pathway remains to be fully defined in shrimp.

In conclusion, our findings elucidate a mechanism by which the commensal microbiota activates the Pvf1/Pvr4/FoxO feedback loop to establish microbiota homeostasis. The hemolymph microbiota is responsible for the activation of FoxO, which leads to the expression of antimicrobial factors and the Pvf1/Pvr4 system. The Pvf1/Pvr4 system enhances PI3K/Akt activity, which prevents FoxO nuclear localization and, thus, its transcriptional activity. This feedback loop balances the FoxO-mediated antimicrobial immunity at a controlled level, which is essential for the persistence and homeostasis of microbiota (Fig 6G). Therefore, this study provides new insights into host-microbiota interactions and arthropod immune regulation, and offers a basis for developing new approaches to disease control in shrimp aquaculture.

## Materials and methods

### Ethics statement

All animal experiments were approved by the Animal Ethics Committee of the Shandong University School of Life Sciences (permit number: SYDWLL-2021–98).

### Animals and microorganisms

Healthy Kuruma shrimp (*M. japonicus*; ~ 8 g) obtained from an aquaculture farm in Jimo, Shandong, China, were cultured in aerated seawater at 22 °C in the laboratory, and fed commercial diets daily. *V. parahaemolyticus* isolated from the shrimp intestinal tract was cultured in Luria-Bertani (LB) liquid medium (3% NaCl, 1% tryptone, and 0.5% yeast extract) at 30 °C overnight. The concentration of *Vibrio parahaemolyticus* used for infection was 1 × 10^6^ CFU/mL.

### Antibiotics treatment

To generate a hemolymph microbiota-depleted (HM-depleted) shrimp model, each shrimp was administered 50 μL of antibiotics mixture containing ampicillin (25 mg/mL), kanamycin (25 mg/mL), and streptomycin (25 mg/mL). The control shrimp group received an equal volume of phosphate-buffered saline (PBS; 140 mM NaCl, 2.7 mM KCl, 10 mM Na_2_HPO_4_, 1.8 mM KH_2_PO_3_, pH 7.4). Antibiotic or PBS injections were administered twice at a 12-h interval.

The effectiveness of microbiota depletion was assessed 48 h after the second injection. Hemolymph samples were plated onto LB (1% tryptone, 0.5% yeast extract, 1% NaCl, 1.5% agar powder), 2216E (2.4% sea salt, 0.5% tryptone, 0.1% yeast extract, 0.01% FeCl_3_, 1.5% agar powder) and TCBS (LA3170; Solarbio, Beijing, China) agar plates and cultured at 30 °C for 24 h to evaluate the clearance efficiency of antibiotics treatment for culturable bacteria. In parallel, hemolymph-derived genomic DNA was extracted, and total bacterial abundance was quantified via qPCR targeting 16S rRNA (S1 Fig). Following the second antibiotics treatment, the shrimp were subjected to a 48-hour starvation period to allow for the host’s metabolism of the antibiotics. All subsequent experiments were performed 48 h after completion of antibiotic treatment.

### Transcriptomic analysis (RNA-seq)

Transcriptome sequencing was conducted to identify the differentially expressed genes (DEGs) between phosphate-buffered saline (PBS)-treated and antibiotics-treated shrimp. Total RNA was extracted from shrimp hemocytes collected 3 d after treatment using TRIzol Reagent (15596–026, Invitrogen, Carlsbad, CA, USA). Each group consisted of at least 30 animals. The experiments were conducted in biological triplicate. Extracted RNA was separated into two portions. One portion was used for transcriptome analysis, whereas the other was used for quantitative real-time reverse transcription polymerase chain reaction (qRT-PCR) validation.

The transcriptome was commercially sequenced using Biomarker Technologies (Beijing, China). The purity, concentration, and integrity of RNA were determined using a Bioanalyzer 2100 system (Agilent, Santa Clara, CA, USA). Acceptable RNA integrity was defined as a value of > 8.0. mRNAs were isolated from 0.75 μg of total RNAs and were inverted into cDNA. Sequencing was conducted on the Illumina platform (San Diego, CA, USA). The raw reads were filtered using Cutadapt and in-house perl script, mapped with the kuruma shrimp genome (GenBank GCA_017312705.1) using HISAT2 and passed through StringTie to obtain transcripts. Bioinformatic analysis was conducted using BMKCloud (www.biocloud.net). Gene expression was compared across samples using the fragments per kilobase per transcript per million mapped reads (FPKM) method. Only genes with FPKM ≥ 2 in the control sample were considered valid. The threshold for differential expression was set at a 2-fold-change, with an adjusted *p*-value ≤ 0.05.

### Plasma preparation and proteomic analysis

The hemolymph from the antibiotics-treated and control shrimp was extracted and combined with an equal volume of cold anticoagulant (450 mM NaCl, 10 mM KCl, 10 mM EDTA, and 100 mM HEPES; pH 7.45), respectively. Each group comprised at least 30 animals. To remove hemocytes, the hemolymph-anticoagulant mixture were centrifuged at 800 × g for 8 min at 4 °C. The supernatant was collected and subsequently was subjected to ultracentrifugation at 140,000 × g for 3 h at 4 °C to remove most accumulated hemocyanin, which was primarily located at the bottom of the lower tube following a previously described method [4]. The supernatant constituted the prepared plasma solution, which was stored at -80 °C. Bradford protein assay kit (C503031, Sangon Biotech) and Coomassie Brilliant Blue staining were used to determine and normalize the plasma protein concentration. The plasma solution was then divided into two parts. One portion was subjected to label-free whole protein quantitative analysis using PTM Biolabs (Zhejiang, China), whereas the other portion was reserved for western blotting validation.

For proteomic analysis, plasma proteins were treated with trypsin at a ratio of 1:50 (trypsin:protein, m/m) through overnight incubation to produce numerous peptides, which were subsequently analyzed by liquid chromatography-tandem mass spectrometry (LC-MS/MS). The peptides were separated using the EASY-nLC 1200 ultra-high-performance liquid phase system, injected into an NSI ion source for ionization, and then analyzed using Orbitrap Exploris 480 MS. Raw MS data were obtained using a data-dependent scan (DDA) program. The experiment was conducted using Proteome Discoverer software, version 2.4.1.15. The search database used was BLAST_Penaeus_japonicus_27405_NCBI_GCF_017312705.1_Mj_TUMSAT_v1.0_20220711.FASTA. The peak intensities and areas of the corresponding peptides in the samples were compared to quantify the proteins. Subsequently, quality control evaluation was conducted to identify and exclude any potential outliers from the dataset, ensuring the reliability of the subsequent analysis. A two-tailed Student’s t-test was used to assess discrepancies in the protein levels between the two groups. Proteins exhibiting differential expression were identified when the *p*-value was ≤ 0.05, and the fold-change was > 1.3.

### Quantitative real-time reverse transcription PCR (qRT-PCR)

Quantitative reverse transcription polymerase chain reaction (qRT-PCR) was used to ascertain the expression levels of *Pvf1*, *Pvr4*, and *LysC* under investigation using AceQ qPCR SYBR Green Master Mix (Q131-03, Vazyme, Nanjing, China). *β-actin* was detected simultaneously as the internal reference. The cDNA template is obtained following similar to the above-described procedures. The specific primers used in the polymerase chain reaction (PCR) program are as follows: The program commenced with a 95 °C incubation for 10 minutes, followed by 40 cycles of 95 °C for 15 s, 60 °C for 50 s, and a plate reading at 72 °C for 2 s. Subsequently, a melting period was initiated, spanning the temperature range of 65–95 °C. The data was processed using the 2^-ΔΔCT^ method. The primers used are listed in S1 Table.

### Western blotting

Plasma proteins were isolated using the previously described plasma preparation methods. The hemocytes were collected following an 8-minute centrifugation at 800 × g at 4 °C, after which they were resuspended and homogenized in PBS. One hundred micrograms of protein were combined with loading buffer and incubated for 10 min at 100 °C. The mixture was then subjected to sodium dodecyl sulfate-polyacrylamide gel electrophoresis (SDS-PAGE) on a 12% gel (304–01, Vazyme). The separated proteins were transferred onto nitrocellulose membranes using a semi-dry transfer protocol on a Jim-X Semi-Dry Blotter (Jim-X; Dalian, China). The membranes were blocked with 5% skim milk in Tris-buffered saline (TBS; 150 mM NaCl, 10 mM Tris-HCl, pH 8.0) for 30 min at room temperature and then incubated with specific primary antibodies for 3 h at room temperature or overnight at 4 °C. Subsequently, the membranes were incubated with secondary antibodies for 3 h at room temperature. This was followed by three washes with TBST (TBS with Tween-20) and an additional wash with TBS. Immunoreactive protein bands were visualized using a high-signal enhanced chemiluminescence (ECL) western blotting substrate (180–5001, Tanon, Shanghai, China) and the Tanon 5200 Chemiluminescence Imaging System.

Antibodies against Pvf1, FoxO, and LysC were used at a dilution of 1:200. Akt antibodies (WL01652; Wanleibio, Liaoning, China) and β-actin antibodies were used at a 1:1,000 dilution. Akt-p antibodies (AP0637, ABclonal, Wuhan, China) were used at a dilution of 1:2,000. Histone H3 antibodies (17168-AP-1; ProteinTech, Rosemont, IL, USA), His-tag antibodies (TA-02; Zhongshan Biotech, Beijing, China), horseradish peroxidase (HRP)-conjugated goat anti-rabbit antibodies (ZB-2301; Zhongshan Biotech) and HRP-conjugated goat anti-mouse antibodies (ZB-2305; Zhongshan Biotech) were used at a 1:5,000 dilution. All antibodies were diluted in 5% skimmed milk.

### Yeast two-hybrid (Y2H) assay

Y2H assay was conducted to ascertain the protein interactions between Pvf1 and Pvr4 using the Matchmaker Gold Yeast Two-Hybrid System (630495, Clontech, Mountain View, CA, USA). The cDNA of the Pvf1 mature peptide and the Pvr4 extracellular domain gene fragment were cloned into pGADT7 prey and pGBKT7 bait vectors, respectively. The corresponding primers are listed in S1 Table. The recombinant vectors encoding bait and prey proteins were co-transformed into Y2H gold-competent cells. Transformants exhibiting positive results were selected using DDO (Leu-, Trp-) medium and subsequently transferred to selective QDO (Ade-, His-, Leu-, Trp-, with or without X-gal) medium. This interaction was identified by the emergence of blue colonies in the selective medium.

### Pull-down assay

A pull-down assay was performed to ascertain the interaction between Pvf1 and Pvr4. The recombinant GST-Pvf1 and His-Pvr4 proteins were expressed and purified according to the following established protocols for protein recombinant expression. The recombinant GST-Pvf1 or GST-tagged control proteins (as bait) were individually mixed with His-Pvr4 in binding buffer, with each protein used at 100 μg. After incubation at 4 °C for six hours with gentle rotation, 100 μL of GST resin (C600031; BBI, Shanghai, China) was added to the mixture, which was subsequently incubated at 4 °C for 45 min. Next, the mixture was centrifuged at 600 × g for 2 min to pellet the resin, and the supernatant was discarded. The resin was resuspended in PBS and centrifugated at 600 × g for 2 min to remove any residual unbound proteins. After six washes, 50 μL of elution buffer was added to the resin to elute the interacting proteins. Protein-protein interactions in the eluate were detected using SDS-PAGE and Coomassie Brilliant Blue staining.

### Immunocytochemical analysis

An immunocytochemical assay was conducted to determine the subcellular localization of FoxO. Hemolymph was extracted using a cold anticoagulant containing 4% paraformaldehyde, which was then fixed for 10 min. The sample was then centrifuged at 800 × g for 8 min at 4 °C and the resulting hemocytes were collected. The hemocytes were then washed with PBS and distributed onto glass slides coated with poly-L-lysine. The slides were then washed three consecutive times with PBS. Subsequently, 0.2% Triton X-100 diluted in PBS was added to the slides and incubated for 10 min. The slides were washed thrice with PBS and blocked with 3% bovine serum albumin (BSA) in PBS at 37 °C for 1 h. FoxO antibodies (1:100 diluted in 3% BSA) were added to the slides and incubated overnight at 4 °C. Following three washes with PBS, goat anti-rabbit Alexa Fluor 488 (A23220, Abbkine, Wuhan, China; 1:1,000 diluted in 3% BSA) was added and incubated for 2 h in the dark. Nuclei were stained with 4’,6-diamidino-2-phenylindole (DAPI) (AS-83210, AnaSpec, San Jose, CA, USA) for 10 min. Subsequently, the slides were washed with PBS for observation and images were captured using a Zeiss LSM 900 confocal microscope (Zeiss, Oberkochen, Germany). The images were subsequently analyzed using ZEN software (Zeiss).

The co-localization of FoxO and nucleus was quantified using three randomly selected fields of view, with a total of more than 100 cells analyzed. Image J software was used to analyze the co-localization between DAPI (nucleus) and IgG DyLight 488 (FoxO) fluorescence signal. The number of co-localized cells was calculated. Statistics were defined as [the hemocytes of FoxO-nuclei colocalization/ all hemocytes observed] × 100%.

### Deposition of recombinant protein (rPvf1) on cell membrane

The detection of rPvf1 on the cell membranes was conducted using immunofluorescence. Twenty-four hours after the injection of siRNA into shrimp, hemocytes were collected and placed on slides following similar to the above-described procedures. Following the attachment of hemocytes to the slide, rTag or rPvf1 (20 μg) was added and incubated for 12 h. Thereafter, the slides were washed thrice with PBS. His-tag antibodies (TA-02, Zhongshan Biotech; 1:200 dilution in 3% BSA) were added to the slides and incubated overnight at 4 °C. Following three washes with PBS, goat anti-mouse IgG DyLight 488 (A23210, Abbkine; 1:1,000 diluted in 3% BSA) was added and incubated for two hours in the dark. Next, the slides were washed three times with PBS. The cell membrane red fluorescent probe Dil (C1036, Beyotime; 1:1,000 dilution in PBS) was then added and incubated for 15 min at 37 °C. Next, the slides were washed thrice with PBS, after which DAPI was added to stain the nuclei for 10 min. Subsequently, the slides were washed with PBS for observation and images were captured using a Zeiss LSM 900 confocal microscope (Zeiss, Oberkochen, Germany). The images were subsequently analyzed using ZEN software (Zeiss).

The co-localization of His-tagged protein and cell membrane was quantified using three randomly selected fields of view, with a total of more than 100 cells analyzed. Image J software was used to analyze the co-localization between Dil (cell membrane) and IgG DyLight 488 (His-tagged protein) fluorescence signal, and rTag was used as the control protein. The number of co-localized cells was calculated. Statistics were defined as [the hemocytes of His tag-membrane colocalization/ all hemocytes observed] × 100%.

### Separation of nuclear and cytoplasmic proteins

To ascertain the distribution of FoxO, nuclear proteins were extracted using a Nuclear Protein Extraction Kit (R0050; Solarbio, Beijing, China) in accordance with the manufacturer’s instructions. Shrimp hemocytes were collected at a specific time (detailed in the legend) and homogenized using cytoplasmic protein extraction reagent containing 1 mM phenylmethanesulfonyl fluoride (PMSF) and a phosphatase inhibitor cocktail (M7528; AbMole Bioscience, Houston, TX, USA). The homogenate was shaken for 20 s and placed on ice for three minutes. Following five cycles of alternating treatment, the homogenate was centrifuged at 13,000 × g for 20 min at 4 °C. Following three washes with PBS, the sediment was resuspended in a nucleoprotein extraction reagent containing 1 mM PMSF and a phosphatase inhibitor cocktail, and processed as previously described. Following a 20-minute centrifugation at 13,000 × g at 4 °C, the supernatant was collected and designated as the nuclear protein extract. Protein concentration was determined using the Bradford protein assay kit. The separated nuclear and cytoplasmic proteins were subjected to quantitative analysis using western blotting. At least five shrimp specimens were used for each sample.

### Chromatin immunoprecipitation (ChIP) assay

To detect the transcriptional regulation of *Pvf1* and *Pvr4* by FoxO, a ChIP assay was performed using a ChIP Assay Kit (P2078, Beyotime). Hemocytes were collected at 3 d after antibiotics treatment and used as a pool for ChIP according to the manufacturer’s instructions. Generally, each treatment collected about 10^6^ hemocytes. Subsequently, hemocytes were washed three times with PBS and resuspended in PBS containing 1% formaldehyde. The suspension was incubated at 37 °C for 10 min to cross-link the target protein with corresponding genomic DNA. Next, 1/10 volume of glycine solution (10 ×) was added to the cross-linking reaction and incubated at 25 °C for 5 min. Then, hemocytes were collected and resuspended in 0.2 mL of SDS Lysis Buffer containing 1 mM PMSF on ice for 10 min to fully lyse hemocytes. In an ice bath, ultrasound (50 W power, 5 s impact, 10 s gap, 14 times in total) was processed to fragment genomic DNA into 200–1000 bp fragments. The suspension was centrifuged at 12,000 × g for 10 min at 4 °C. The resultant supernatant was collected and added ChIP Dilution Buffer to a total volume of 1.5 mL.

Divide the suspension into three parts as input (100 μL), FoxO antibody (700 μL) and IgG antibody which does not cross-react with any shrimp proteins (700 μL) groups. FoxO antibody (5 μg) and IgG antibody were separately added into the corresponding group for shaking incubation at 4 °C overnight after pre-cleared with Protein A + G Agarose/Salmon Sperm DNA (50 μL). Next, Protein A + G Agarose/Salmon Sperm DNA (20 μL) were individually added for shaking incubation for 30 min at 4 °C to enrich antibody-protein-DNA complex. After centrifugation at 1,000 × g for 1 min at 4 °C, the agarose-precipitate was collected and subsequently washed by the Low Salt Immune Complex Wash Buffer, High Salt Immune Complex Wash Buffer, and LiCl Immune Complex Wash Buffer in sequence, and finally twice by TE Buffer. The Elution buffer (1% SDS, 0.1M NaHCO_3_) was used for eluting bound precipitate. After centrifugation at 1,000 × g for 1 min at 4 °C, the supernatant was added with 1/20 volume of 5 M NaCl. The mixture was heated at 65 °C for 4 h for de-crosslinking to obtain immunoprecipitates. Immunoprecipitates were analyzed by RT-PCR using specific primers (S1 Table for fragments containing FoxO-binding sites).

### Electrophoretic mobility shift assay (EMSA)

EMSA was conducted to investigate the interaction between FoxO and the promoter regions of *Pvf1* and *Pvr4*. Firstly, FoxO-binding sites in the promoters of *Pvf1* and *Pvr4* were predicted. Generally, the 5′ end of *Pvf1* and *Pvr4* cDNA were cloned by rapid amplification of cDNA ends (RACE) using the SMARTer RACE 5′ Kit (634868; Clontech, Mountain View, CA), according to the manufacturer’s instructions. The transcription start sites were determined by analyzing and comparing the cDNA sequence and the transcriptome sequencing results. The upstream sequences obtained from the *M. japonicus* genome (GenBank GCA_017312705.1, GCA_002291165.1) were verified using PCR and sequencing. Potential FoxO binding sites were analyzed using the online PROMO 3.0 (alggen.lsi.upc.es/cgi bin/promo_v3) and JASPAR (jaspar.genereg.net) tools. The wild-type and mutated oligonucleotide probes were synthesized (Sangon Biotech, Shanghai, China) and labeled with biotin using an EMSA Probe Biotin Labeling Kit (GS008, Beyotime). Oligonucleotide sequences are listed in S1 Table.

EMSA was conducted using a Chemiluminescent EMSA Kit (GS009; Beyotime), following the methodology outlined by the manufacturer. Purified recombinant FoxO protein (rFoxO, 2 μg) was incubated with wild-type and mutated probes (5 ng) at 25 °C for 20 min in binding buffer. In competition-binding assays, excess unbiotinylated wild-type probes (500 ng) were introduced into the mixture. Next, this samples were analyzed on 6% acrylamide gels in 0.5 × Tris-borate-EDTA buffer, followed by electroblotting onto a nylon membrane. After cross-linking under ultraviolet light for 10 min, the membrane was incubated with HRP-conjugated streptavidin. Labeled DNA probes were detected and visualized using a chemiluminescent biotin-labeled nucleic acid detection kit.

### Protein recombinant expression

Sequences encoding the mature peptides of Pvf1 were amplified and ligated into the pET32a(+) and pGEX4T-2 plasmids. Sequences encoding the full-length FoxO protein and the extracellular domain protein of Pvr4 were amplified and ligated into the pET30a(+) plasmid. The specific primers used for the amplification of the aforementioned genes are listed in S1 Table. The recombinant plasmids were transformed into *Escherichia coli* Rosetta (DE3) strains for expression under the control of the isopropyl-β-D-thiogalactopyranoside (IPTG) induction system at 28 °C for 6 h. The Pvf1-pET32a(+) plasmid, named rPvf1, was expressed and formed in the inclusion bodies. Recombinant plasmids encoding FoxO and Pvr4 were expressed as soluble proteins. The resulting proteins were designated rFoxO and His-Pvr4, respectively. The inclusion bodies were dissolved in a denaturing solution (8 M urea), followed by dialysis with a urea buffer of high to low concentrations for denaturation and refolding, obtaining rPvf1. The soluble proteins rFoxO and His-Pvr4 were purified using Ni-NTA His-binding resin (70666; Merck, Darmstadt, Germany). Affinity chromatography was used to purify GST-Pvf1 expressed from the pGEX4T-2 plasmid. ProteinIso GST resin (DP-201; TransGen Biotech, Beijing, China) was used as the affinity matrix and elution was achieved by adding glutathione. To generate His-Trx (thioredoxin) (rTag) and glutathione-S-transferase (GST) (rGST), empty vectors were used. Endotoxins were removed by washing the column with cold 0.1% Triton X-114 before the final elution. All proteins were subsequently subjected to dialysis in PBS and stored at -80 °C. To examine the effects of overexpression *in vivo*, rPvf1 or the corresponding control rTag was injected into shrimp at a dose of 2 μg.

### Antibody generation and purification

The rPvf1 solution (1 mg/mL, 1.5 mL) and an equal volume of complete Freund’s adjuvant (F5881, Sigma-Aldrich, St. Louis, MO, USA) was used for emulsification. The mixture was administered subcutaneously to New Zealand White rabbits during the initial immunization procedure. The second immunization was conducted 25 d later, with complete adjuvant replacement using an incomplete adjuvant (F5506, Sigma-Aldrich). Rabbit antiserum was collected 7 d later and stored at -80 °C until used in western blotting. The specificity validation of the Pvf1 antibody in S6 Fig. Antisera against FoxO, β-actin, and LysC were generated according to a previously described method [21,22].

### RNA interference (RNAi)

RNA interference (RNAi) was used to suppress gene expression *in vivo* using small interfering RNA (siRNAs). The most appropriate siRNA sequences were identified using an online tool (Biodev.extra.cea.fr/DSIR/DSIR). Two pairs of reverse complementary oligonucleotides containing the T7 promoter were synthesized commercially and annealed to generate the siRNA templates. The corresponding oligonucleotide sequences are presented in S1 Table for reference. To prevent potential off-target effects, siRNA sequences were analyzed against the *M. japonicus* genome (GenBank accession number GCA_017312705.1) using an online BLAST tool (ncbi.nlm.nih.gov/BLAST). siRNA was synthesized using a T7 RNAi Synthesis siRNA Transcription Kit (TR102, Vazyme), and the product was purified by phenol/chloroform/isoamyl alcohol extraction. Following verification by gel electrophoresis and quantification by spectrophotometer, the siRNA was injected into the shrimp hemocoel at a dose of 5 μg/g body weight. At a designated point in the study, total RNA was extracted from hemocytes or intestines to detect gene expression in relation to siRNA.

### Measurement of the abundance of total hemolymph microbiota

Shrimp surfaces were sterilized using a 75% ethanol solution. Hemolymph was collected from the shrimp using anticoagulants. Equal volumes of hemolymph were processed for total DNA extraction using the QIAamp DNA Blood Mini Kit (51104; Qiagen, Hilden, Germany), according to the manufacturer’s instructions. Quantitative polymerase chain reaction (qPCR) targeting the 16S ribosomal RNA gene was conducted to quantify the levels of 16S rDNA using the universal primer pair presented in S1 Table. The colony-forming units (CFUs) of the hemolymph microbiota were obtained by fitting a quantitative standard curve specific to the bacterial strain.

### 16S rDNA high throughput sequencing

Commercial 16S rDNA sequencing was performed by Biomarker Technologies. Total microbial DNA was extracted from shrimp hemolymph at 6 d post *siPvf1* injection. Each sample was prepared using at least 10 individual shrimp, with three biological replicates performed for each experimental group. The quality and quantity of DNA were verified prior to sequencing. Sequencing was performed using Illumina NovaSeq 6000. Effective circular consensus sequence (CCS) data were analyzed using Trimmomatic v0.33, cutadapt 1.9.1 and QIIME2 2020.6 for sequence recognition, filtering, and chimera removal. Reads were clustered into operational taxonomic units (OTUs) at a 97.0% similarity threshold using Matplotlib_1.4.3 software [56]. Taxonomic annotation was performed against the Matplotlib-v1.5.1 reference database, and species abundance tables at different taxonomic levels were generated using Python2 software [57]. The microbial community structure at the phylum and genus level was displayed using R language tools (https://www.rproject.org/).

### Application of inhibitor, agonist and stimulant

MK-2206 2HCl (M1837, AbMole, Houston, USA) is a highly selective Akt1/2/3 inhibitor. LY294002 (HY-10108; MCE, New Jersey, USA) is a pan-Pi3k inhibitor. SC79 (HY-18749, MCE) is an Akt agonist. The inhibitors and agonists were dissolved in phosphate-buffered saline (PBS) containing 5% dimethyl sulfoxide (DMSO) and 40% polyethylene glycol 300 (PEG 300) and then injected into the shrimp according to the IC50 dosage. An equal quantity of DMSO. Subsequent experimental manipulations were conducted two hours after the injection. A peptidoglycan-derived stimulant (muropeptides) dissolved in water and administered to shrimp at a dosage of 1 μg per shrimp. An equal volume of water was then added to the control group.

### Histological analysis

Histological analysis was conducted to ascertain the tissue morphology. The hepatopancreas were collected and fixed using Davidson’s AFA fixative, which comprises 30% ethanol, 22% formalin, and 11.5% acetic acid. Following a 24-hour fixation period, the tissue was subjected to dehydration, embedded in paraffin, and sectioned at a thickness of 7 μm. Subsequently, the tissues were stained with hematoxylin and eosin (H&E). The slides were observed under a BX51 microscope (Olympus, Tokyo, Japan) and images were captured using a DP70 digital camera system (Olympus).

### Statistical analysis

The data obtained from the survival assay were analyzed using the log-rank (Mantel-Cox) test in GraphPad Prism 8 software (GraphPad Inc., La Jolla, CA, USA). The remaining data were analyzed using the Student’s *t-test*, and a significant difference was accepted at *p* < 0.05.

## Supporting information

S1 Fig Establishment of a model with antibiotic-induced hemolymph microbiota depletion in shrimp. (A) Elimination of hemolymph culturable bacteria by antibiotics treatment. Shrimp were administered 50 μL of antibiotics, while the control shrimp received an equal volume of PBS. At 48 h post-administration, shrimp hemolymph was collected and plated separately onto LB, 2216E and TCBS agar plates. Plates were cultured at 30˚C for 24 h, and hemolymph culturable bacteria were determined using plated-counting method. (B) Elimination of hemolymph total bacteria by antibiotics treatment. Shrimp hemolymph was collected after 48 h antibiotics treatment. Hemolymph-derived genomic DNA were extracted and performed total bacterial load assessment via qPCR quantification of 16S rRNA. ***p < 0.001.
(TIF)

S2 Fig Validation of recombinant Pvf1 and its rescue effect on hemolymph microbiota homeostasis. (A) Recombinant Pvf1 (rPvf1) and control tag (rTag) proteins. rPvf1 mature peptide and Tag plasmid were transformed into *E. coli* Rosetta (DE3) for induced expression and protein purification and analyzed by SDS-PAGE and Coomassie Brilliant Blue staining. (B) The blotting assay of rPvf1 and rTag in hemolymph. Shrimp were injected with rPvf1 or rTag, and equal volumes of hemolymph were collected at the indicated time points. The presence of the recombinant proteins was detected by blotting assay using an anti-His-tag antibody. (C) Total bacterial abundance in the hemolymph after *Pvf1* knockdown followed by rPvf1 supplementation. Shrimp were injected with rPvf1 (2 μg) after *Pvf1* silencing, while control shrimp received an equal amount of rTag. Hemolymph was collected 3 and 6 d, and total DNA was extracted, and bacterial load was quantified by qPCR targeting 16S rDNA. Bacterial abundance was calculated as colony-forming units per milliliter hemolymph (CFU/mL) based on a standard curve. In scatter plots, each dot represents one shrimp. ***p < 0.001 and ns, not significant.
(TIF)

S3 Fig Regulatory effect of Pvf1 on PGN-induced FoxO nuclear localization. (A) Enhancement of PGN-induced FoxO nuclear localization after *Pvf1* knockdown. Shrimp were injected with PGN after siRNA treatment. Hemocytes were collected 6h after PGN injection and subjected to immunocytochemical analysis. Scale bar = 10 μm. FoxO nuclear localization was quantified from three randomly selected fields using ImageJ software. ***p < 0.001.
(TIF)

S4 Fig The regulatory effect of Pvf1 on PGN-induced FoxO transcriptional activity in HM-depleted shrimp. (A-B) Induction of LysC expression and FoxO nuclear localization in hemocytes by Pvf1 specific antibodies (anti-Pvf1). Antibiotics were used to generate hemolymph microbiota-depleted (HM-depleted) shrimp. After treatment with antibiotics and replenishment with PGN, shrimp were injected with anti-Pvf1 to neutralize the effect of natural Pvf1, whereas the control group was injected with IgG that recognized no shrimp protein. *LysC* mRNA levels at 12 h (A) and FoxO nuclear levels at 6 h (B) are shown.
(TIF)

S5 Fig *V. parahaemolyticus* enhanced the expression of Pvf1 and Pvr4. (A-B) Temporal mRNA expression profiles of *Pvf1* and *Pvr4* in hemocytes after *V. parahaemolyticus* challenge. The shrimp were soaked in seawater containing *V. parahaemolyticus*. At the indicated time points, the total RNA was extracted for qRT-PCR analysis. (C) Protein levels of Pvf1 in hemocytes were analyzed by western blotting after *V. parahaemolyticus* challenge. (D) Plasma Pvf1 levels were analyzed using western blotting after *V. parahaemolyticus* infection. (E-F) Inhibition of *V. parahaemolyticus*-induced *Pvf1* and *Pvr4* expression in hemocytes following *FoxO* knockdown. Presilenced shrimp were stimulated with *V. parahaemolyticus*. The mRNA expression of *Pvf1* and *Pvr4* at 12 h. (G) Blotting assay of Pvf1 in hemocytes of *V. parahaemolyticus-*challenged FoxO presilenced shrimp. (H) Inhibition of *V. parahaemolyticus*-induced plasma Pvf1 levels by *FoxO* knockdown. ***p < 0.001 and ns, not significant.
(TIF)

S6 Fig Specificity validation of the Pvf1 antibody. (A) The blotting assay for validation of Pvf1 antibody specificity.
(TIF)

S1 Table Primers and probes used in this study. (DOCX)
