# Supplementary figures and images for: Feedback coordination of FoxO-mediated antibacterial immunity by PDGF/VEGF signaling establishes hemolymph microbiota homeostasis in shrimp
Source: PLoS Pathog. 2026 Jun 4;22(6):e1014307. doi: 10.1371/journal.ppat.1014307 (PMC13252836; doi:10.1371/journal.ppat.1014307)

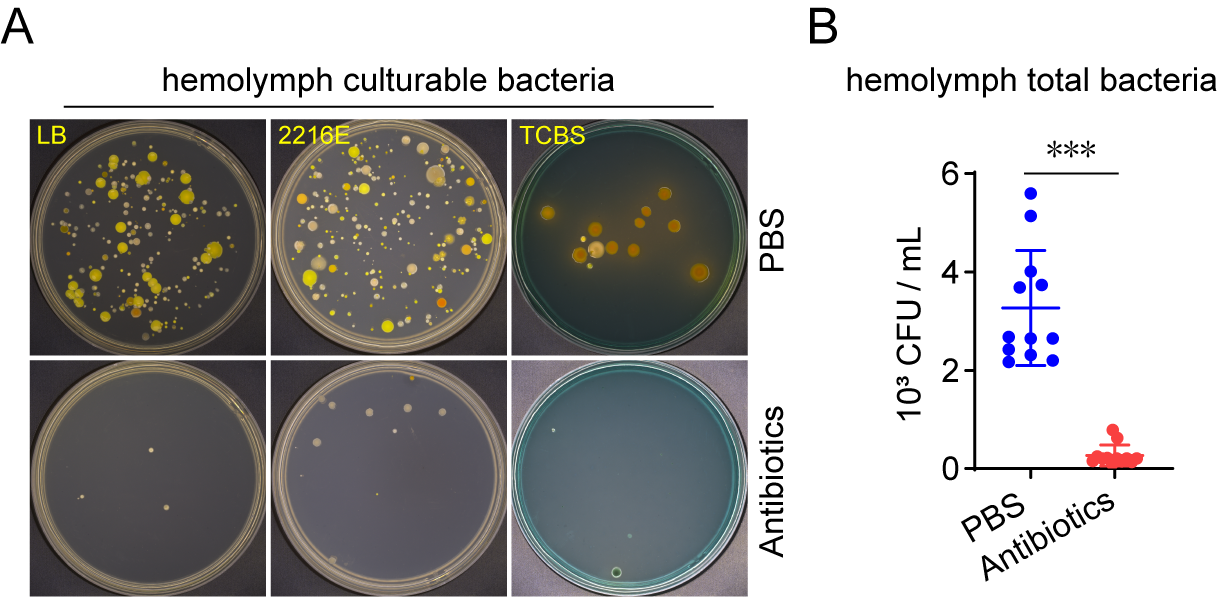

Supplement: S1 Fig — Shrimp were administered 50 μL of antibiotics, while the control shrimp received an equal volume of PBS. At 48 h post-administration, shrimp hemolymph was collected and plated separately onto LB, 2216E and TCBS agar plates. Plates were cultured at 30˚C for 24 h, and hemolymph culturable bacteria were determined using plated-counting method. (B) Elimination of hemolymph total bacteria by antibiotics treatment. Shrimp hemolymph was collected after 48 h antibiotics treatment. Hemolymph-derived genomic DNA were extracted and performed total bacterial load assessment via qPCR quantification of 16S rRNA. ***p < 0.001. (TIF) [file ppat.1014307.s001.tif]

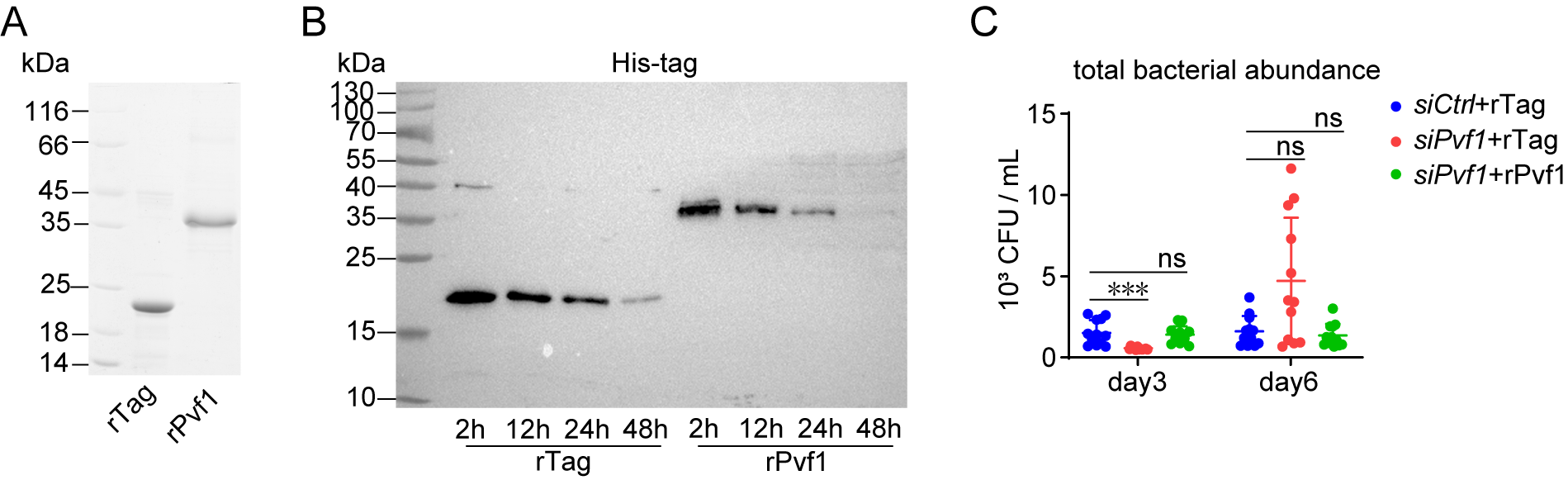

Supplement: S2 Fig — (A) Recombinant Pvf1 (rPvf1) and control tag (rTag) proteins. rPvf1 mature peptide and Tag plasmid were transformed into E. coli Rosetta (DE3) for induced expression and protein purification and analyzed by SDS-PAGE and Coomassie Brilliant Blue staining. (B) The blotting assay of rPvf1 and rTag in hemolymph. Shrimp were injected with rPvf1 or rTag, and equal volumes of hemolymph were collected at the indicated time points. The presence of the recombinant proteins was detected by blotting assay using an anti-His-tag antibody. (C) Total bacterial abundance in the hemolymph after Pvf1 knockdown followed by rPvf1 supplementation. Shrimp were injected with rPvf1 (2 μg) after Pvf1 silencing, while control shrimp received an equal amount of rTag. Hemolymph was collected 3 and 6 d, and total DNA was extracted, and bacterial load was quantified by qPCR targeting 16S rDNA. Bacterial abundance was calculated as colony-forming units per milliliter hemolymph (CFU/mL) based on a standard curve. In scatter plots, each dot represents one shrimp. ***p < 0.001 and ns, not significant. (TIF) [file ppat.1014307.s002.tif]

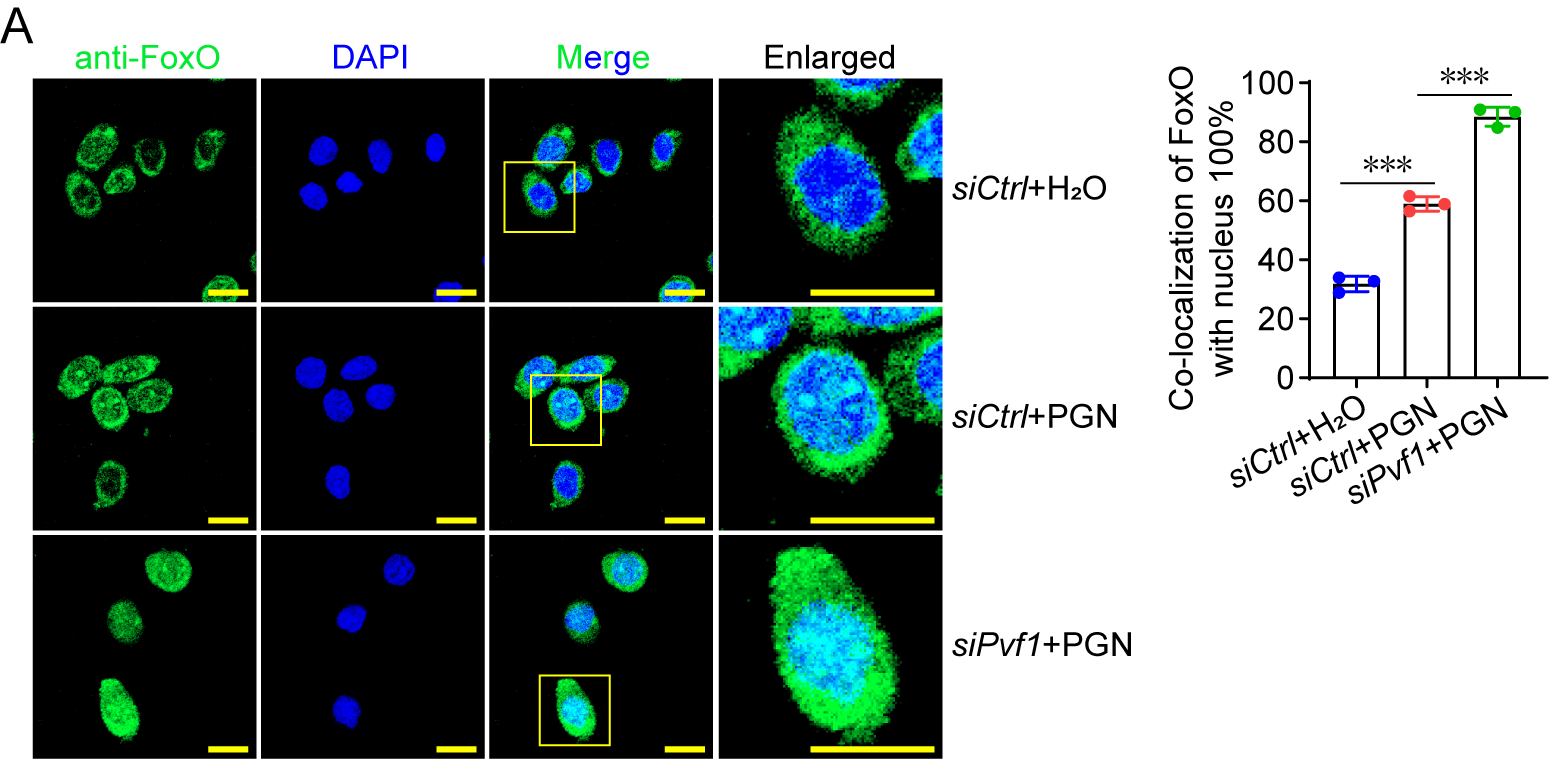

Supplement: S3 Fig — (A) Enhancement of PGN-induced FoxO nuclear localization after Pvf1 knockdown. Shrimp were injected with PGN after siRNA treatment. Hemocytes were collected 6h after PGN injection and subjected to immunocytochemical analysis. Scale bar = 10 μm. FoxO nuclear localization was quantified from three randomly selected fields using ImageJ software. ***p < 0.001. (TIF) [file ppat.1014307.s003.tif]

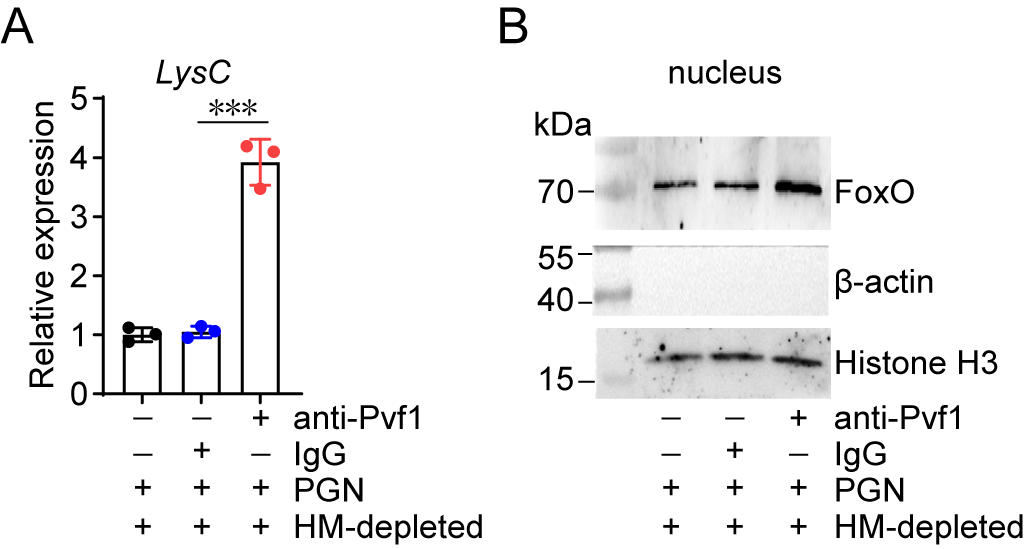

Supplement: S4 Fig — (A-B) Induction of LysC expression and FoxO nuclear localization in hemocytes by Pvf1 specific antibodies (anti-Pvf1). Antibiotics were used to generate hemolymph microbiota-depleted (HM-depleted) shrimp. After treatment with antibiotics and replenishment with PGN, shrimp were injected with anti-Pvf1 to neutralize the effect of natural Pvf1, whereas the control group was injected with IgG that recognized no shrimp protein. LysC mRNA levels at 12 h (A) and FoxO nuclear levels at 6 h (B) are shown. (TIF) [file ppat.1014307.s004.tif]

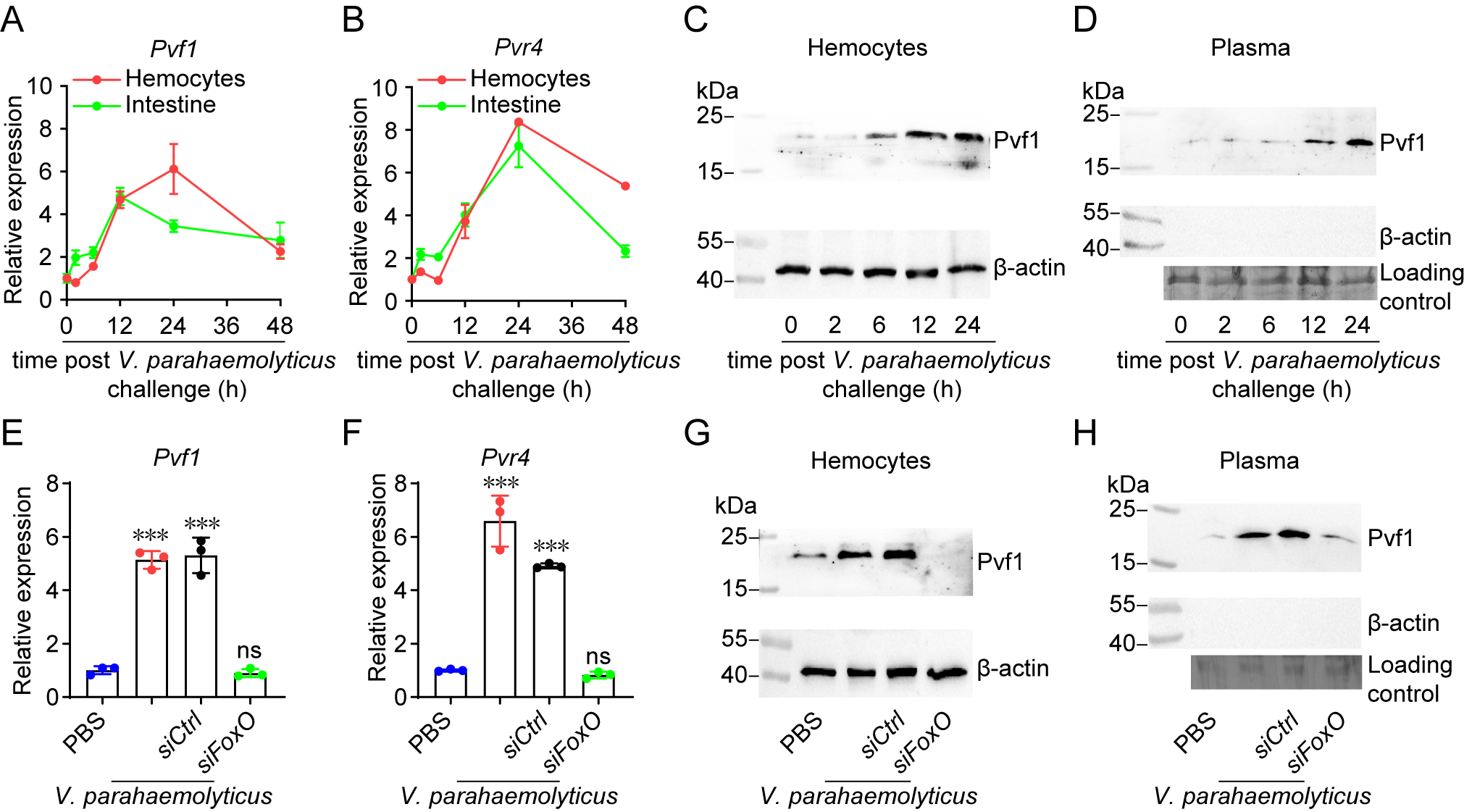

Supplement: S5 Fig — (A-B) Temporal mRNA expression profiles of Pvf1 and Pvr4 in hemocytes after V. parahaemolyticus challenge. The shrimp were soaked in seawater containing V. parahaemolyticus. At the indicated time points, the total RNA was extracted for qRT-PCR analysis. (C) Protein levels of Pvf1 in hemocytes were analyzed by western blotting after V. parahaemolyticus challenge. (D) Plasma Pvf1 levels were analyzed using western blotting after V. parahaemolyticus infection. (E-F) Inhibition of V. parahaemolyticus-induced Pvf1 and Pvr4 expression in hemocytes following FoxO knockdown. Presilenced shrimp were stimulated with V. parahaemolyticus. The mRNA expression of Pvf1 and Pvr4 at 12 h. (G) Blotting assay of Pvf1 in hemocytes of V. parahaemolyticus-challenged FoxO presilenced shrimp. (H) Inhibition of V. parahaemolyticus-induced plasma Pvf1 levels by FoxO knockdown. ***p < 0.001 and ns, not significant. (TIF) [file ppat.1014307.s005.tif]

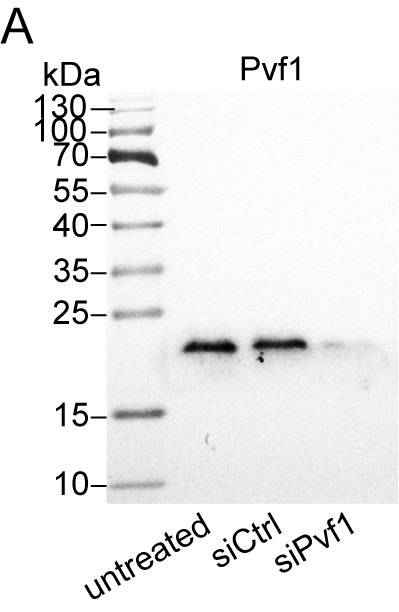

Supplement: S6 Fig — (A) The blotting assay for validation of Pvf1 antibody specificity. (TIF) [file ppat.1014307.s006.tif]
