# Supplementary material for: Feedback coordination of FoxO-mediated antibacterial immunity by PDGF/VEGF signaling establishes hemolymph microbiota homeostasis in shrimp
Source: PLoS Pathog. 2026 Jun 4;22(6):e1014307. doi: 10.1371/journal.ppat.1014307 (PMC13252836; doi:10.1371/journal.ppat.1014307)
Supplement: S1 Table — (DOCX) [file ppat.1014307.s007.docx]

**S1 Table. Primers and probes used in this study**

| Primers | Sequence (5’-3’) |
| --- | --- |
| **(q)RT-PCR** |  |
| Pvf1RTF | GCAGTGAGTCGCTGTTCTAT |
| Pvf1RTR | TTCAACGGGGATTTCTAAG |
| Pvr4RTF | TACGATAAGCACATCCGACC |
| Pvr4RTR | TGGACCACTACGAAGGCG |
| LysCRTF | ATTTGCGGAGTTGCTGGAG |
| LysCRTR | GGAATTGGAAGGGCGAGAC |
| 16S rRNARTF | ACTCCTACGGGAGGCAGCAGT |
| 16S rRNARTR | TATTACCGCGGCTGCTGGC |
| β-actinRTF | CAGCCTTCCTTCCTGGGTATGG |
| β-actinRTR | GAGGGAGCGAGGGCAGTGATT |
| **RNAi** |  |
| Pvf1Oligo1 | GATCACTAATACGACTCACTATAGGGAGGCGCTCAGTCGGTTACAAGAAATT |
| Pvf1Oligo2 | AATTTCTTGTAACCGACTGAGCGCCTCCCTATAGTGAGTCGTATTAGTGATC |
| Pvf1Oligo3 | AAAGGCGCTCAGTCGGTTACAAGAAATCCCTATAGTGAGTCGTATTAGTGATC |
| Pvf1Oligo4 | GATCACTAATACGACTCACTATAGGGATTTCTTGTAACCGACTGAGCGCCTTT |
| Pvr4Oligo1 | GATCACTAATACGACTCACTATAGGGGGATAAAGAAGAAGTAGAAAGTT |
| Pvr4Oligo2 | AACTTTCTACTTCTTCTTTATCCCCCTATAGTGAGTCGTATTAGTGATC |
| Pvr4Oligo3 | AAGGATAAAGAAGAAGTAGAAAGCCCTATAGTGAGTCGTATTAGTGATC |
| Pvr4Oligo4 | GATCACTAATACGACTCACTATAGGGCTTTCTACTTCTTCTTTATCCTT |
| FoxOOligo1 | GATCACTAATACGACTCACTATAGGGCGATATTATGGAAACTACACCTT |
| FoxOOligo2 | AAGGTGTAGTTTCCATAATATCGCCCTATAGTGAGTCGTATTAGTGATC |
| FoxOOligo3 | GATCACTAATACGACTCACTATAGGGGGTGTAGTTTCCATAATATCGTT |
| FoxOOligo4 | AACGATATTATGGAAACTACACCCCCTATAGTGAGTCGTATTAGTGATC |
| CtrlOligo1 | GATCACTAATACGACTCACTATAGGGCGGAAAGCTCACCCTGAAATTTT |
| CtrlOligo2 | AAAATTTCAGGGTGAGCTTTCCGCCCTATAGTGAGTCGTATTAGTGATC |
| CtrlOligo3 | GATCACTAATACGACTCACTATAGGGAATTTCAGGGTGAGCTTTCCGTT |
| CtrlOligo4 | AACGGAAAGCTCACCCTGAAATTCCCTATAGTGAGTCGTATTAGTGATC |
| **Recombinant expression** |  |
| Pvf1HisF | GCCATGGCTGATATCGGATCCAGGCGATGCTTGACCATCAA |
| Pvf1HisR | GTGGTGGTGGTGGTGCTCGAGTCAGTCCATACATCTGCATGTATTGT |
| Pvf1GSTF | GATCTGGTTCCGCGTGGATCCAGGCGATGCTTGACCATCAA |
| Pvf1GSTR | GTCACGATGCGGCCGCTCGAGTCAGTCCATACATCTGCATGTATTGT |
| Pvr4HisF | GCCATGGCTGATATCGGATCCCAAGATTATTATGAGGAAGAAGTCAAACC |
| Pvr4HisR | GTGGTGGTGGTGGTGCTCGAGGACATTCTTACGACGCCCAGG |
| FoxOHisF | GCCATGGCTGATATCGGATCCATGATGGCAACCAGTTTCTTTTC |
| FoxOHisR | GTGGTGGTGGTGGTGCTCGAGGCGGACCCACTGGTTCCC |
| **Yeast two hybrid** |  |
| Pvf1ADF | GCCATGGAGGCCAGTGAATTCAGGCGATGCTTGACCATCAA |
| Pvf1ADR | CAGCTCGAGCTCGATGGATCCGTCCATACATCTGCATGTATTG |
| Pvr4BDF | ATGGCCATGGAGGCCGAATTCCAAGATTATTATGAGGAAGAAGTCAAACC |
| Pvr4BDR | CCGCTGCAGGTCGACGGATCCGACATTCTTACGACGCCCAGG |
| **ChIP** |  |
| Pvf1ChIPF | AGAAAGGCTAAAAGTATGTG |
| Pvf1ChIPR | CACTTTCTTTATATCTCTCAT |
| Pvr4ChIPF | TTTTGCTCGTTCCCTGA |
| Pvr4ChIPR | CAGCGTCTTATCTTCTTTCACT |
| **EMSA** |  |
| Pvf1WTF | CTAAAAGTATGTGTGTGTTTACAAATCTGTTTCT |
| Pvf1WTR | AGAAACAGATTTGTAAACACACACATACTTTTAG |
| Pvf1MutF | CTAAAAGTATGTGTGCACGCGAGAATCTGTTTCT |
| Pvf1MutF | AGAAACAGATTCTCGCGTGCACACATACTTTTAG |
| Pvr4WTF | GGGCACGATTGCTTGAAAGCAACA TTCGTCGCATGCA |
| Pvr4WTR | TGCATGCGACGAATGTTGCTTTCAAGCAATCGTGCCC |
| Pvr4MutF | GGGCACGATTGCTGTGCACGCGAGTTCGTCGCATGCA |
| Pvr4MutF | TGCATGCGACGAACTCGCGTGCACAGCAATCGTGCCC |
